# A Customized Bayesian Algorithm to Optimize Enzyme-Catalyzed Reactions

**DOI:** 10.1021/acssuschemeng.3c02402

**Published:** 2023-08-03

**Authors:** Ryo Tachibana, Kailin Zhang, Zhi Zou, Simon Burgener, Thomas R. Ward

**Affiliations:** † Department of Chemistry, University of Basel, Mattenstrasse 24a, BPR 1096, CH-4058, Basel, Switzerland; ‡ National Center of Competence in Research (NCCR) “Molecular Systems Engineering”, 4058 Basel, Switzerland; § National Center of Competence in Research (NCCR) “Catalysis”, ETHZ, 8093 Zurich, Switzerland

**Keywords:** Bayesian optimization, catalytic reaction, design of experiment, enzyme reaction, response surface methodology

## Abstract

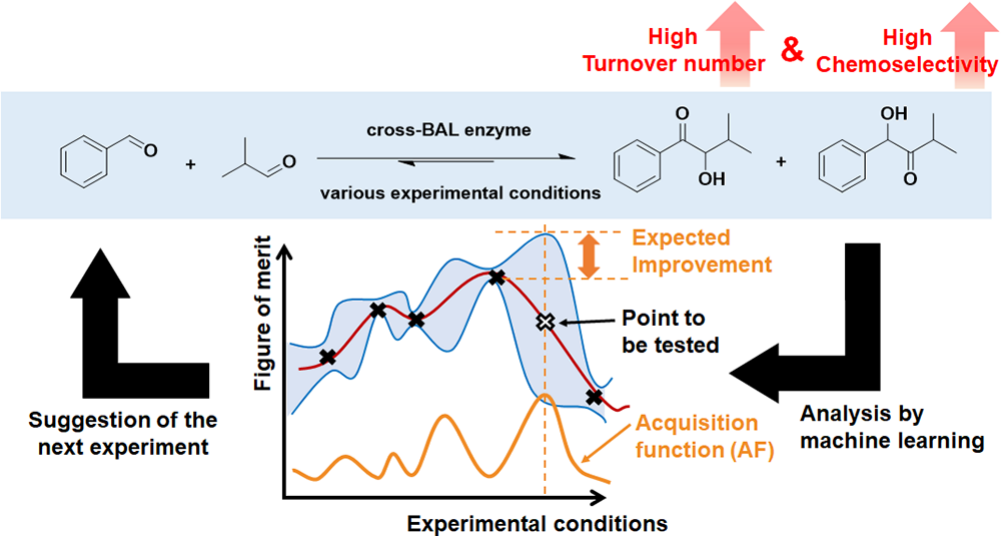

Design of experiments (DoE) plays an important role in optimizing the catalytic performance of chemical reactions. The most commonly used DoE relies on the response surface methodology (RSM) to model the variable space of experimental conditions with the fewest number of experiments. However, the RSM leads to an exponential increase in the number of required experiments as the number of variables increases. Herein we describe a Bayesian optimization algorithm (BOA) to optimize the continuous parameters (e.g., temperature, reaction time, reactant and enzyme concentrations, etc.) of enzyme-catalyzed reactions with the aim of maximizing performance. Compared to existing Bayesian optimization methods, we propose an improved algorithm that leads to better results under limited resources and time for experiments. To validate the versatility of the BOA, we benchmarked its performance with biocatalytic C–C bond formation and amination for the optimization of the turnover number. Gratifyingly, up to 80% improvement compared to RSM and up to 360% improvement vs previous Bayesian optimization algorithms were obtained. Importantly, this strategy enabled simultaneous optimization of both the enzyme’s activity and selectivity for cross-benzoin condensation.

## Abstract 2

A machine learning-based search algorithm is reported that maximizes the efficiency and selectivity of enzyme reactions while reducing experimental costs.

## Introduction

**Figure 1:**
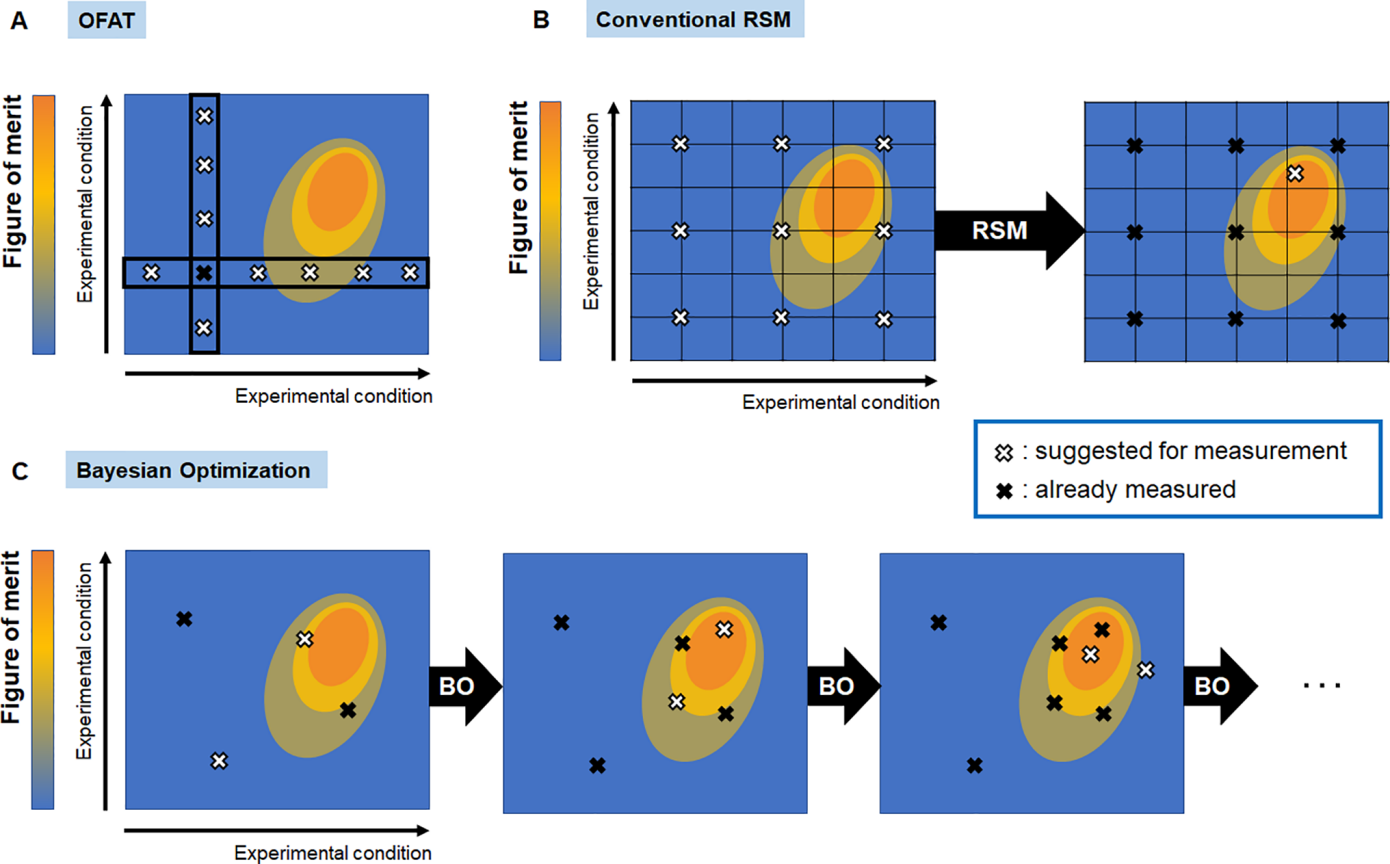
Comparison of the sampling process. (A) In OFAT, one variable of the reaction conditions is selected and optimized, while the other parameters are held constant. By iterating this process, the variables are changed one by one. (B) In the RSM, the entire hyperspace of reaction conditions is sampled homogeneously. Then, analysis of the single-iteration leads to the prediction of the optimized reaction conditions for a given FoM. Accordingly, regions of hyperspace leading to suboptimal FoM are sampled unnecessarily. (C) In Bayesian optimization, experimental sampling and prediction are iteratively repeated. For each iteration, the reaction conditions are suggested for which the FoM is predicted to be best. Accordingly, the better the resultant hyperspace, the denser the sampling density is applied.

The optimization of experimental reaction conditions to maximize the figures of merit of a catalytic reaction (FoM, i.e. yield, turnover number, selectivity, rate, etc.) plays an essential role both in academic and industrial settings.^1^ The outcome of a chemical reaction is governed by a complex network of interactions between reactants, catalysts, solvent, and other ingredients, as well as temperature, pressure, pH, etc. To maximize a given FoM, multiple variables need to be optimized. Since most variables influence each other, it is challenging to determine the global optimum by optimizing individual variables one by one (i.e., one-factor-at-a-time, OFAT, Figure 1A).^2,3^ When considering interactions among variables, the number of experiments required for optimization increases exponentially as the number of parameters increases. Therefore, in a majority of catalysis optimization campaigns, it is impossible to comprehensively evaluate all possible combinations of experimental variables.

To address this challenge, design of experiments (DoE)^4,5^ has been used extensively as a methodology to scrutinize the correlation between factors and experimental results with the fewest number of experiments possible in a search for improved FoMs.^2,6−8^ One of the most commonly used methods in DoE is the response surface methodology (RSM).^2,9,10^ This method computes a response surface model (relying on an approximate equation) between the experimental conditions and FoMs, and then improves the accuracy of this model through a limited number of experiments. Various strategies have been proposed to reduce the number of experiments, such as Box-Behnken design,^11,12^ central composite design,^13,14^ etc.^15^ Using a software package, the experimenter can automatically select the best of these strategies and apply DoE without any need for in-depth prior knowledge of the mechanism.^2^

However, RSM remains challenging as the number of experiments grows exponentially with a linear increasing number of variables. If the amount of data is insufficient for the complexity of the model, then the optimized model will be inaccurate. In order to model the complex relationship among multiple variables, it is necessary to closely examine the influence of each factor on the FoM through preliminary experiments and to create experimental tables to identify the most important parameters. This task requires a detailed understanding of RSM, making it challenging to apply it correctly for scientists with limited knowledge of the tool. To overcome some of these challenges, definitive screening designs have been introduced as a method to model a surface response.^16,17^ One of the reasons for the massive increase in the experimental cost is that the sampling points are selected nearly homogeneously from the entire parameter hyperspace. Indeed, the RSM is a single-iteration optimization process, whereby all experiments are performed simultaneously to determine the optimal parameters. As the process relies on a single iteration, all regions of the parameter hyperspace are sampled without bias. This procedure thus can lead to sampling portions of the parameter hyperspace that are inherently not worth exploring (Figure 1B).

**Figure 2:**
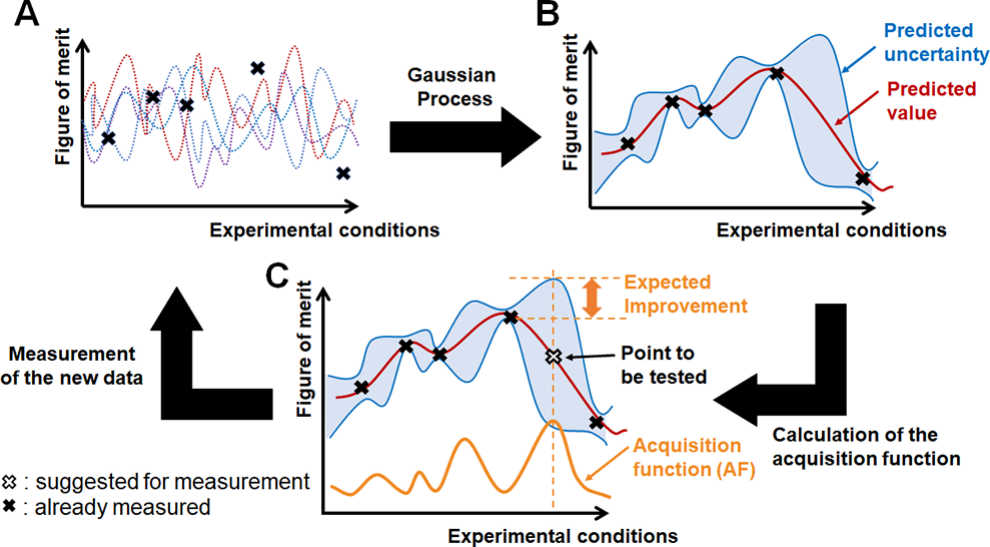
Overview of the Bayesian optimization algorithm (BOA). This iterative process is repeated until the figure of merit (FoM) reaches a target value. (A) An initial data set (black crosses) is experimentally acquired, enabling the algorithm to build a model. As a fitting curve, various types of functions are assumed (dotted lines), and their likelihood is computed by Bayesian inference. (B) A Gaussian-process regression computes the predicted FoM value (solid red line) and its uncertainty (blue area) for untested experimental conditions. (C) By applying the acquisition function (AF, orange line), the experimental conditions predicted to improve FoM most (white cross) are computed. The FoM for these experimental conditions are (i) computed (white cross), (ii) validated experimentally, and (iii) added to the existing experimental data set. The process is iterated until the FoM reaches a target value.

In recent years, Bayesian optimization algorithms, based on machine learning, have been applied toward the optimization of experimental conditions.^18−21^ In this method, the Gaussian process regression^22,23^ first predicts the FoM value and its uncertainty under untested reaction conditions. From these data and with the aim of improving the FoM, the algorithm computes which conditions should be experimentally tested. For this purpose, an evaluation formula called the acquisition function (AF) is applied. In the evaluation of the FoM, two types of strategies are applied: exploration and exploitation. (1) Exploration of areas that have not yet been tested and (2) exploitation of good conditions that have already been obtained. By iteratively repeating the process of prediction and experiments, regions with greater potential for an improved FoM can be preferentially explored, enabling a more efficient optimization (Figure 1C). In addition, in a Gaussian process regression, a virtually infinite dimension of functions is assumed as a model, and the likelihood of the model functions is computed based on Bayes’ theorem, according to the existing data.^23^ Compared with the conventional RSM whose fitting function is limited to a few dimensions, it is expected that Bayesian optimization can identify complex relationships among variables under experimental conditions. Also, it does not require a preparative process as RSM, and it is easy to implement even without expertise in DoE. Importantly, the complexity of the underlying function used in Bayesian optimization need not be understood to enable experimentalists to apply Bayesian algorithms to the optimization of enzyme-catalyzed-reactions. While such algorithms can provide an answer, they do not supply any explanation concerning the highly complex relationship between the reaction parameters subjected to optimization. The Gaussian process regression and Bayesian optimization are expected to have potential applications in a variety of fields such as flow chemistry,^24,25^ automatic chemical design,^26^ adjustment of machine learning systems^27,28^ or experimental devices,^29^ materials engineering,^30^ biological engineering,^31,32^ etc. However, there is still a limited number of examples of this technology applied to enzyme reaction development, despite the potential applicability of Gaussian process regressions.^33−36^ Since the correlation between turnover numbers (the figure of merit) and experimental conditions is typically rather continuous, Bayesian optimization using Gaussian processes is expected to provide a particularly accurate experimental design. In this study, we evaluated the performance of RSM and Bayesian optimization to optimize an FoM for two enzyme-catalyzed reactions. In the process, we identified limitations of the acquisition function used in existing Bayesian optimization^19^ and adjusted it. The resulting Bayesian optimization algorithm (BOA) led to the rapid identification of significantly improved FoMs for enzyme-catalyzed reactions. The BOA workflow that we applied in this study is outlined in Figure 2. Initially, the FoM and experimental variables to be optimized are identified, and initial experimental data are collected from either experiments or the literature (A).

In a Gaussian-process regression, the fitting curve is not fixed via a single type of equation, but various functions are assumed as stochastically valid. Based on the experimental data, the Bayesian inference computes predicted values for the FoM and their uncertainties for untested experimental conditions (B). An AF is computed to identify the values of the experimental conditions, which should be tested in the next iteration (C). The data point that possesses the highest value of the AF is selected, and the resulting experimental data resulting from these conditions are added to the data set (return to A). Then, the Gaussian process-regression and the evaluation of the AF are performed again (B, C). This iterative process is repeated until a targeted FoM is reached. As more data are collected, the model is refined and becomes more accurate: the probability of identifying improved experimental conditions increases accordingly. In an ideal Bayesian optimization, only the single point with the largest AF for each iteration is evaluated, thus significantly slowing the iterative optimization process. To expedite the procedure, we adopted a batch optimization process to evaluate a handful of reaction conditions during each iteration. The Kriging believer algorithm^37^ was selected as batch optimization method with reference to previous research.^19^ In this method, when the second and subsequent experimental conditions to be tested are identified, Gaussian-process regression is performed including conditions identified in the previous iterations (Figure S1).

## Results and Discussion

**Scheme 1:**
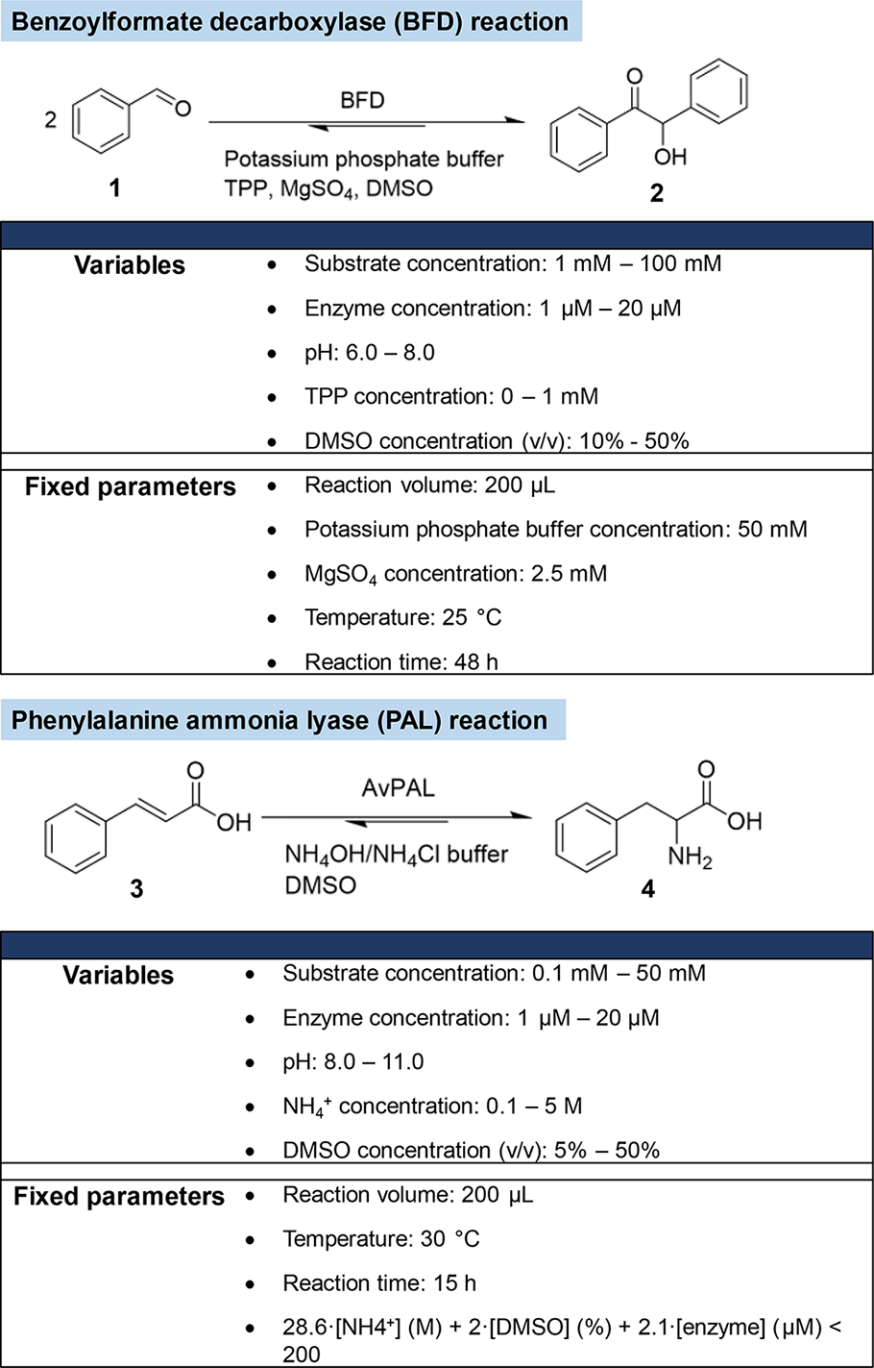
Selected Enzyme-Catalyzed Reactions Used to Benchmark and Improve the Performance of the DoE Algorithms

Initially, we compared the performance of MODDE (a commercial program based on RSM) and with a Bayesian optimization method based on the reported system.^19^ Assuming that the DoE is performed by a scientist with no expertise with the optimization software, the screening was set up as follows: only the reaction scheme and the range of experimental conditions to be explored were defined as initial conditions. No preliminary study of the FoM response was carried out. The experimental conditions to be evaluated were identified solely by using the automated process provided by the software: no intuition, prior knowledge, or preliminary screening was required. We selected two widely used enzyme catalyzed reactions: (i) a carboxy-lyase reaction, catalyzed by benzoylformate decarboxylase (BFD),^38−40^ and (ii) the conversion of *trans*-cinnamic acid and ammonia to phenylalanine, catalyzed by phenylalanine ammonia lyase (PAL).^41−45^ The optimization was performed with the aim of maximizing the total turnover number (TON), used as the FoM. Five parameters were selected as variables for both the PAL- and the BFD-catalyzed reactions (Scheme 1).

In enzymatic reactions, enzyme and substrate concentrations are directly related to the determination of the TON, and pH has a significant effect on enzyme activity. For both reactions, DMSO was used as a cosolvent to increase the solubility of the substrate in the aqueous reaction medium. All other factors described in Scheme 1, including buffer concentration, temperature, reaction volume, and reaction time were kept constant throughout the DoE.

### BFD Reaction

In addition to the setting above, the concentration of thiamine pyrophosphate (TPP) was selected as a variable. For some TPP-dependent enzymes, high TPP concentrations can be inhibiting.^46^ Accordingly, the TPP concentration plays a critical role in maximizing the TON with the purified enzymes. Based on the experimental table generated by MODDE, 29 experiments were performed (Tables S1 and S2). The contour plot for the BFD reaction at a TPP concentration of 1 mM is displayed in Figure S2. As can be appreciated, the response surface increases monotonically with an increasing substrate concentration and decreasing enzyme concentration. The activity of the enzyme tends to decrease with higher concentrations of DMSO, and the BFD performs best at pH 8. With the help of the program, optimal experimental conditions were proposed. Gratifyingly, when the BFD reaction was performed using the predicted best conditions, a TON = 3289 was obtained, significantly higher than the TON = 2776 predicted by MODDE for these experimental conditions (Tables 1 and S3).

**Table 1:**
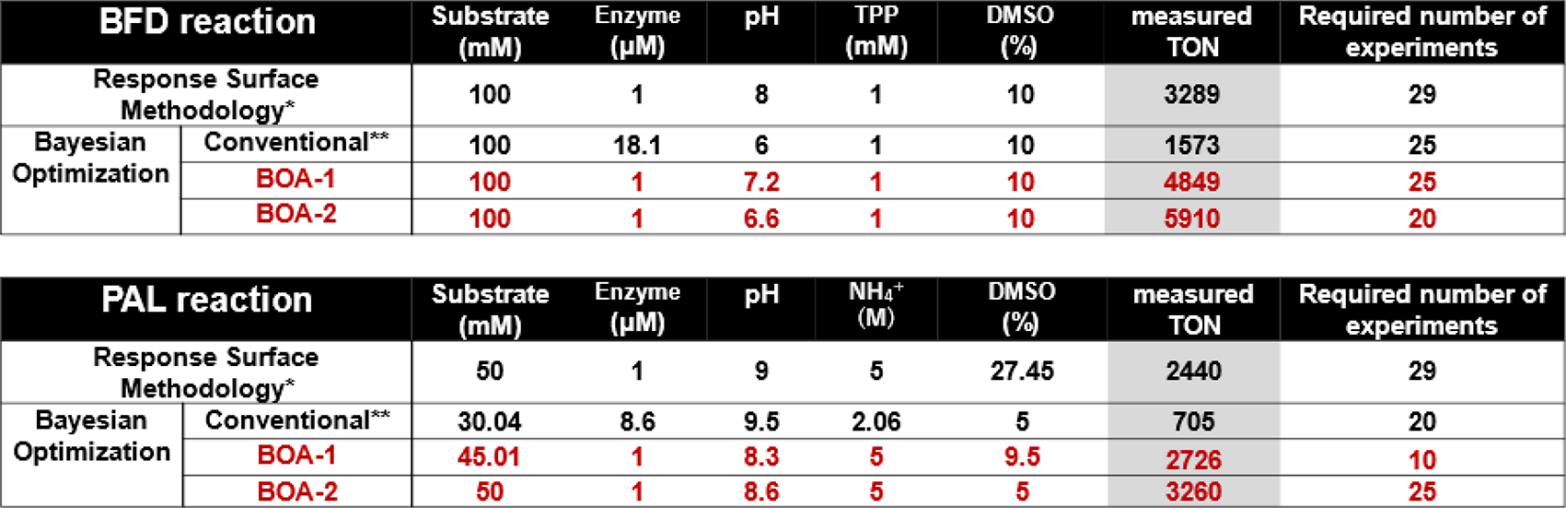
Comparison of the Optimized Reaction Conditions Determined for the BFD and PAL-Catalyzed Reactions Using DoE Algorithms^a^

a *MODDE®, **Bayesian optimization with conventional AF and BOA.

### PAL Reaction

The NH_4_^+^ concentration was selected as a variable as it acts as a cosubstrate for the hydroamination of *trans-*cinnamic acid. The solubility of the reagents resulted in a limit on the total amount of NH_4_^+^, DMSO, and the enzyme that could be evaluated. This is reflected in the equation 28.6·[NH_4_^+^] (M) + 2·[DMSO] (%) + 2.1·[enzyme] (μM) < 200 (see the table in Scheme 1). In the same way as the BFD reaction, experimental tables and predicted optimal conditions were prepared (Tables S2 and S4). The response surfaces in the presence of 5% DMSO are displayed in Figure S3. As expected, the enzyme performance is poor at low NH_4_^+^ concentrations. At pH 8, a low enzyme concentration combined with a high substrate concentration results in a high TON. However, the PAL reaction under the predicted optimal conditions affords a TON = 1544, which is significantly lower than the predicted TON = 2764 (Table S4).

For both reactions, the generated response surfaces are quite monotonous and do not fully capture complex interactions such as the relationship between pH, DMSO concentration, and substrate solubility. We surmise that this contributes to reducing the reliability of the final predictions.

Next, a Bayesian optimization based on a conventional setting, derived from the recently reported EBDO model^19^ (see Supporting Information for details), was performed, starting with five initial reaction conditions randomly selected. We performed 5 experiments per cycle and anticipated identifying better conditions than those obtained in the RSM with fewer experiments.

**Figure 3:**
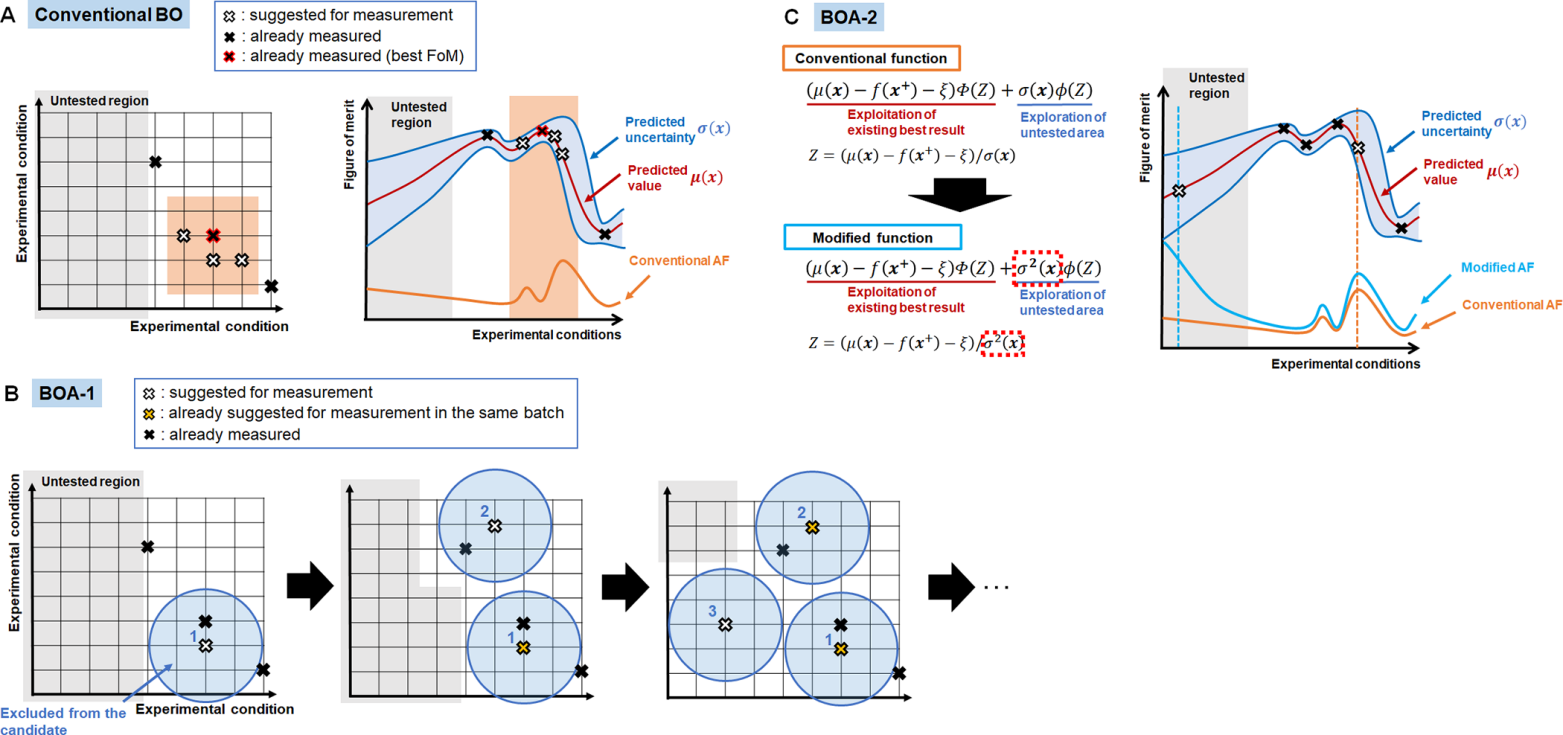
Modification of the algorithm and AF in BOA-1 and BOA-2. In the conventional method (A), the prediction leans toward the exploitation of experimental conditions resulting in high FoM within the existing data set (red cross) and neglects sampling the unexplored regions of the experimental hyperspace (gray area). In BOA-1 (B), when determining the next experimental conditions to test, points close to the previously evaluated conditions (in the blue disk) are excluded. This method avoids concentrating the suggested points in the same area during one iteration. In BOA-2 (C), the AF is modified. By squaring the standard deviation of the predictions, σ2(*x*), the function is expected to give more emphasis to regions far away from the existing data, which have a high uncertainty. It enables the full exploration of the entire hyperspace spanned by the experimental conditions.

Unexpectedly, the optimization process led to the identification of optimized reaction conditions, resulting in significantly lower TON compared to the RSM described above: TON = 1573 (for BFD) and 705 (for PAL). The five reaction conditions proposed for each iteration of optimization turned out to be similar to each other and concentrated around the reaction conditions previously explored (Tables S5 and S6). Inspection of the AF, eq 1, reveals the following trends of the algorithm: (i) it leans strongly toward the exploitation of experimental conditions resulting in high FoM within the existing data set, and (ii) it is reluctant to sample the unexplored regions of the experimental hyperspace (Figure 3A). We hypothesized that this AF might be challenged to identify the optimum global optimum condition. To address this issue, we considered modifying the AF and the corresponding sampling algorithm used in the Bayesian optimization. As can be appreciated from eq 1, the AF includes an evaluation term for (i) the exploration of untested regions and (ii) the exploitation of the best result in the existing data. With a rapid optimization process in mind, it is indispensable to maintain a balance between these two factors. Various formulas have been used to address this delicate balance including: upper/lower confidence bound,^47^ probability of improvement,^47,48^ and expected improvement.^48,49^ Inspired by the previous report,^19^, we selected the expected improvement function (EI).
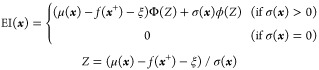
1where *f*(**x**^*+*^) is the current best data, *μ*(**x**) is the predicted mean value, *σ*(**x**) is the predicted standard deviation, Φ and ϕ represent the cumulative distribution function and the probability density function of standard normal distribution, and ξ is a parameter to determine the balance between exploration and exploitation, which is set to 0.01. The expected improvement function computes how much the FoM will improve compared to the best FoM resulting from the experimental data. Shields et al. reported that this function enables the efficient optimization of chemical reactions.^19^

To overcome the challenge of overemphasizing exploitation vs exploration^50^ observed with both BDF- and PAL-catalyzed reactions (Tables S5 and S6), we sought to implement a mechanism that favors a more active exploration of the untested experimental regions. With this goal in mind, we evaluated two complementary strategies (i.e., BOA-1 and BOA-2 respectively) to balance the exploration/exploitation components of the AF.

For BOA-1, the algorithm used to determine the next round of experimental conditions was modified, while keeping the AF unchanged. In the batch optimization process, the second and subsequent proposed experimental conditions are selected among points that are sufficiently distant from the previously sampled conditions. Specifically, for each grid point generated by the program, the minimum distance to an existing data point is computed, and the program automatically eliminates grid points that are within the threshold value, which is set 1.0 on standardized scales (see Figure 3B for a pictural representation of the BOA-1 sampling strategy to restrict the search area and the Supporting Information for the Python code for the detailed process). This strategy avoids accumulating experimental conditions located in the same area during the same iteration. In this way, it is expected that a globally optimal solution can be reached more rapidly. For BOA-2, the algorithm used to determine the next round of experimental conditions was kept, and the expected improvement function was modified, as illustrated in Figure 3C. By squaring the standard deviation of the predictions, σ^2^(**x**), the experimental values in areas of higher uncertainty are given higher priority. This favors a selection of experimental conditions to be tested that are farther from the existing data points. In practice, in early BOA iterations, as the amount of data is small, there are many points characterized by large uncertainties. Such points gain additional scores in the search value by squaring their uncertainties. This increases the probability of being selected as a point to be tested in the next iteration. As the amount of data increases, the number of points with large uncertainties is reduced. The effect of squaring the uncertainties to promote exploration is gradually reduced, ultimately reaching the original expected improvement. Accordingly, the exploitation weight increases in later stages of BOA optimization. In applying this function, FoM values in the existing data set were scaled so that the maximum value is 10 to prevent the predicted uncertainty from becoming too large.

The results of BOA-1 and BOA-2 for both BDF- and PAL-catalyzed reactions are also compiled in Table 1 (entries 3 and 4), and Tables S7–S10. For both enzymatic reactions, BOA-1 and BOA-2 afforded higher TONs than either RSM and the conventional Bayesian optimization. Moreover, the number of required experiments tended to be lower for BOA. In the case of BOA-1, the highest FoM from the RSM was matched in the second iteration already, ultimately leading to the identification of significantly improved TONs after the five iterations systematically applied in this study (Tables S7 and S8). In the latter half of the optimization, when fine-tuning of parameters was required, the search efficiency was reduced by the limitation that closely related experimental conditions were not proposed by the BOA during this iteration.

**Scheme 2:**
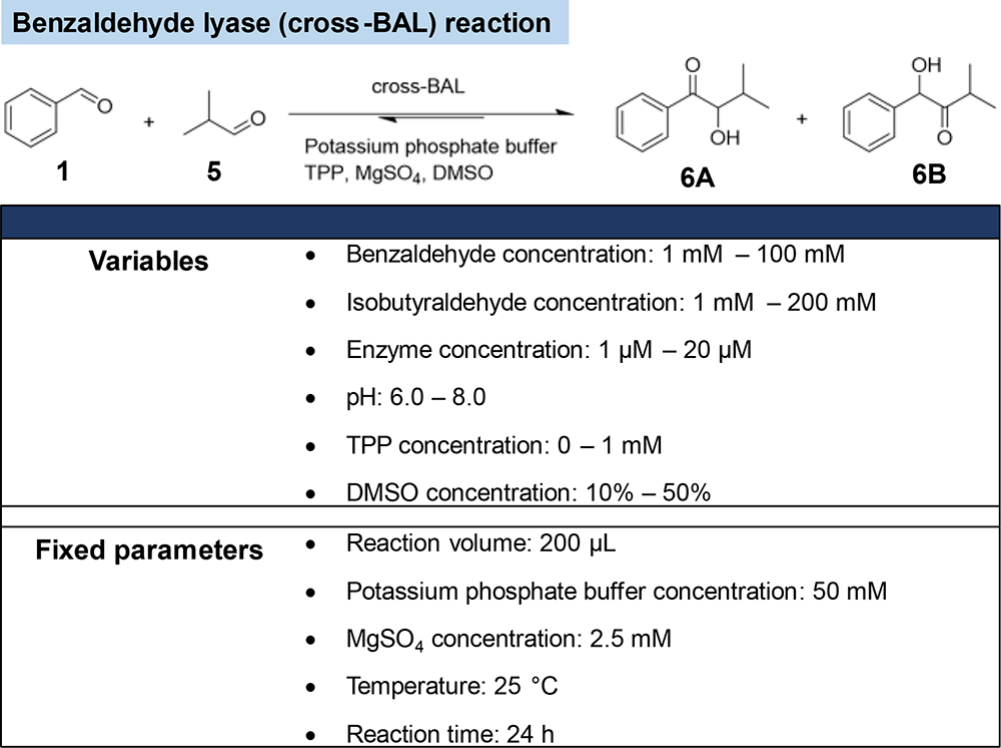
Benzaldehyde Lyase As a Testbed for the Simultaneous Optimization of Two Figures of Merit: Total Turnover Number and Chemoselectivity

For example, in the BFD reaction, the optimal solution for RSM was reached in the second iteration, but subsequent fine-tuning of the pH required three additional iterations to ultimately achieve the highest TON. In contrast, the BOA-2 achieved high efficiency without limiting the search range (Tables S9 and S10). In the first iteration, experimental conditions were selected mainly at the boundaries of the search range for each variable. This enables examining trends in the entire variable hyperspace. In the latter part of the optimization, variables were fine-tuned while remaining in the high-FoM region. It reveals that the exploration of the untested region and exploitation of existing best results is well balanced. Based on these observations, the BOA-1 tends to be more effective during the early optimization stage and the BOA-2 in the late optimization stage. Having identified promising BOAs, we set out to test these toward the optimization of two FoMs simultaneously. Maximizing multiple FoMs simultaneously is still a difficult and important challenge, and DoE methods targeting this goal are receiving a lot of attention.^51−54^ With this goal in mind, we selected the benzaldehyde lyase carboligation between benzaldehyde and isobutyraldehyde (cross-BAL, Scheme 2).^55^

Two α-hydroxyketones may result from cross-carboligation. While the enantioselectivity of both products mostly exceeds 92% ee, the ratio of the yield of these products is 1:1, leaving plenty of room for the improvement of chemoselectivity.^55^ We thus set to optimize simultaneously both chemoselectivity and TON. Considering the example of the expected improvement modification for optimization under constraints with a second FoM value,^56^ the AF was modified to identify experimental conditions whereby the chemoselectivity (FoM 2) is expected to be greater than a set threshold, eq 2.
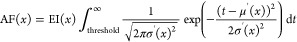
2where EI(*x*) is the function used in BOA-1 or -2, μ′(*x*) and σ′(*x*) are the predicted mean value and predicted standard deviation for FoM 2. The threshold is the target value of FoM 2 (i.e., chemoselectivity). This value should be set by weighing the relative importance of the two FoMs: the higher the threshold value for FoM 2, the less accurate the prediction of the main FoM 1 will be. For this validation, we set a higher emphasis on the TON (i.e., FoM 1) aimed at reaching *a* > 70% chemoselectivity, corresponding to *a* > 85:15 ratio of isomers.

The number of variables in the experimental conditions was increased from the two previous examples from five to six. This resulted in a larger experimental table for optimization by RSM and required 47 experiments (Table S11). In general, the higher the TON, the lower the chemoselectivity. There were, however, a few experimental conditions for which both FoMs were improved simultaneously. The best score was TON = 1426 and 94% chemoselectivity. However, the optimal reaction conditions predicted by the software based on these measured data did not lead to improved FoM (Table S12). The response surfaces created for each of the TON and chemoselectivity reveal very different values for the six parameters to achieve their respective maxima (Figures S4 and S5). This highlights the difficulty of optimizing both FoMs simultaneously using the RSM method. In contrast, the results obtained using BOA-1 and BOA-2 outperformed RSM while requiring only 10–25 experiments—vs 47 for the RSM. The maximum TON is 5441 with a chemoselectivity = 90.5% (Tables 2, 3, S13, S14).

**Table 2:**
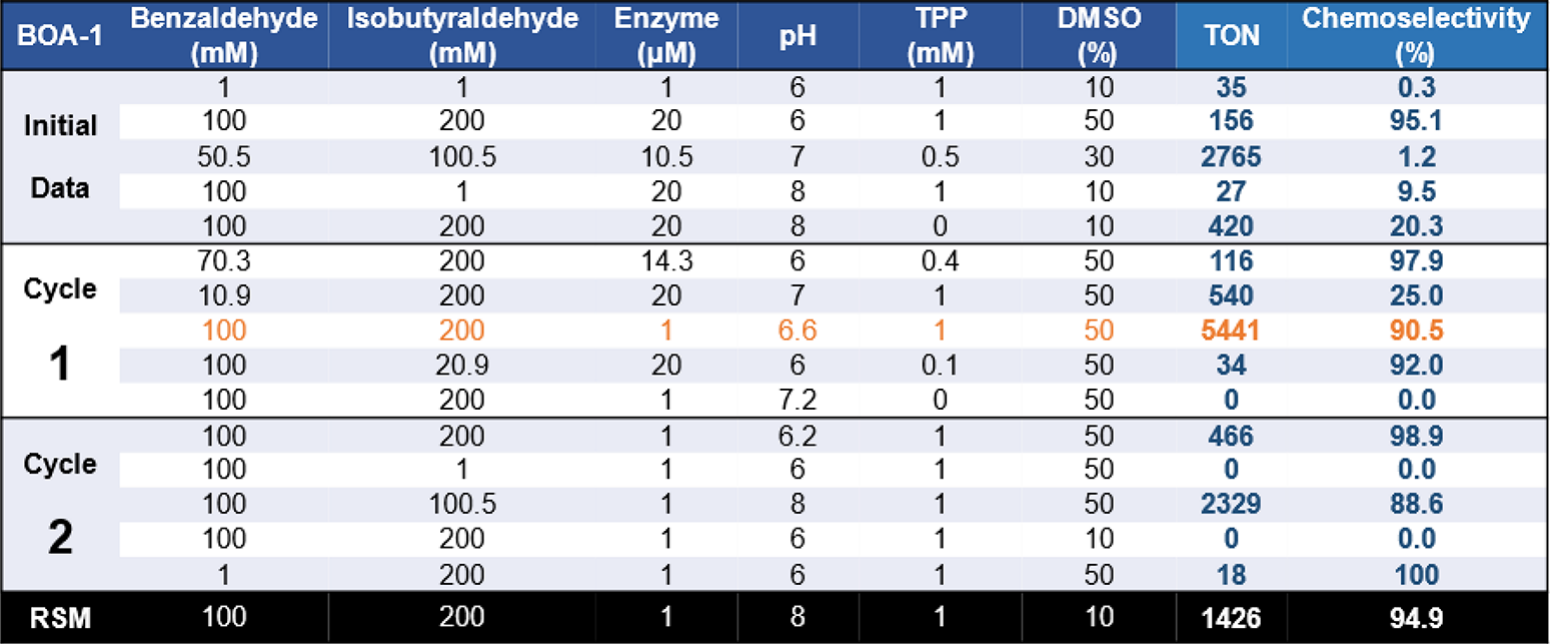
Summary of the Iterative Optimization Process for the Cross-BAL-Catalyzed Reaction between Benzaldehyde and Isobutyraldehyde Using BOA-1

**Table 3:**
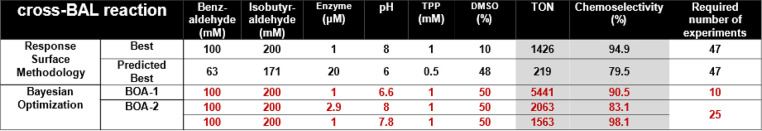
Comparison of the Optimized Reaction Conditions Resulting from DoE Based on the RSM, BOA-1, and BOA-2 for the Cross-BAL-Catalyzed Reaction

For both BOA-1 and BOA-2, the algorithms succeeded in identifying the optimal conditions for RSM within a smaller number of experiments and then reached even better conditions through further rounds of iterations. From these results, we suggest that Bayesian optimization is more efficient than RSM for the optimization of enzyme-catalyzed reactions. The RSME of the model also suggests that Bayesian optimization is more flexible in understanding the relationship between experimental conditions and FoMs (Table S15). Importantly, the BOA can accurately capture how the efficiency of an enzymatic reaction of interest is affected by changes in experimental conditions without requiring any prior experiments or knowledge of the relationship between the experimental conditions and the FoMs. Moreover, the modifications that we introduced in the AF enable addressing the exploration-exploitation bias encountered in conventional Bayesian optimization.^19^ BOA-1 favors more extensive exploration within a single iteration. Thus, it is more likely to yield valuable data in the early stages of optimization. BOA-2 provides effective suggestions by balancing the exploration of unknown regions with the exploitation of existing results. Importantly, for the three examples presented herein, the modified acquisition function implemented in BOA-2 is effective. This suggests that it may be widely applicable for the optimization of diverse enzymatic catalyzed transformations. In future work, we will compare the performance of the BOAs presented herein with recently reported Bayesian algorithms (TSEMO, Par-EGO, etc).^57,58^ The python scripts used can be downloaded from GitHub (see Supporting Information).

As highlighted in the last example, whereby two FoMs are optimized simultaneously, the method based on Bayesian optimization can be adapted to the optimization of complex and highly interdependent experimental conditions. By tailoring the AF and algorithm that determines the condition to be tested, each FoM can be optimized in the required evaluation method. This method can be applied not only to reactions with purified enzymes but also to nonenzyme-mediated chemical reactions, reactions that span multiple processes, and the reactions in complex systems such as cells. We anticipate that straightforward control of the experimental conditions based on the BOA will reduce the cost of optimizing experimental conditions, which can be the rate-limiting step in the development of chemical reactions and the engineering of biological systems.

## Supporting Information Available

DNA sequences (PDF-1,PDF-2, PDF-3)
Supplementary figures, materials, methods, availability of the program, synthesis protocols (PDF-4)

## Abbreviations

AF: acquisition function
BFD: benzoylformate decarboxylase
BOA: Bayesian optimization algorithm
cross-BAL: benzaldehyde lyase
DMSO: dimethyl sulfoxide
DoE: design of experiments
EI: expected improvement
FoM: figures of merit
OFAT: one-factor-at-a-time
PAL: phenylalanine ammonia lyase
RSM: response surface methodology
TON: turnover number
TPP: thiamine pyrophosphate

## References

[ref1] Schwaller P.; Vaucher A. C.; Laplaza R.; Bunne C.; Krause A.; Corminboeuf C.; Laino T. Machine Intelligence for Chemical Reaction Space. WIREs Comput. Mol. Sci. 2022, 12 (5), e1604 10.1002/wcms.1604.

[ref2] Bowden G. D.; Pichler B. J.; Maurer A. A Design of Experiments (DoE) Approach Accelerates the Optimization of Copper-Mediated 18F-Fluorination Reactions of Arylstannanes. Sci. Rep. 2019, 9 (1), 11370 10.1038/s41598-019-47846-6. 31388076 PMC6684620

[ref3] Gilman J.; Walls L.; Bandiera L.; Menolascina F. Statistical Design of Experiments for Synthetic Biology. ACS Synth. Biol. 2021, 10 (1), 1–18. 10.1021/acssynbio.0c00385. 33406821

[ref4] Lee R. Statistical Design of Experiments for Screening and Optimization. Chemie Ing. Technol. 2019, 91 (3), 191–200. 10.1002/cite.201800100.

[ref5] Weissman S. A.; Anderson N. G. Design of Experiments (DoE) and Process Optimization. A Review of Recent Publications. Org. Process Res. Dev. 2015, 19 (11), 1605–1633. 10.1021/op500169m.

[ref6] Murray P. M.; Bellany F.; Benhamou L.; Bučar D.-K.; Tabor A. B.; Sheppard T. D. The Application of Design of Experiments (DoE) Reaction Optimisation and Solvent Selection in the Development of New Synthetic Chemistry. Org. Biomol. Chem. 2016, 14 (8), 2373–2384. 10.1039/C5OB01892G. 26699438

[ref7] Lendrem D. W.; Lendrem B. C.; Woods D.; Rowland-Jones R.; Burke M.; Chatfield M.; Isaacs J. D.; Owen M. R. Lost in Space: Design of Experiments and Scientific Exploration in a Hogarth Universe. Drug Discovery Today 2015, 20 (11), 1365–1371. 10.1016/j.drudis.2015.09.015. 26505134

[ref8] Kasemiire A.; Avohou H. T.; De Bleye C.; Sacre P.-Y.; Dumont E.; Hubert P.; Ziemons E. Design of Experiments and Design Space Approaches in the Pharmaceutical Bioprocess Optimization. Eur. J. Pharm. Biopharm. 2021, 166, 144–154. 10.1016/j.ejpb.2021.06.004. 34147574

[ref9] Chelladurai S. J. S.; Murugan K.; Ray A. P.; Upadhyaya M.; Narasimharaj V.; Gnanasekaran S. Optimization of Process Parameters Using Response Surface Methodology: A Review. Mater. Today Proc. 2021, 37, 1301–1304. 10.1016/j.matpr.2020.06.466.

[ref10] Manmai N.; Unpaprom Y.; Ramaraj R. Bioethanol Production from Sunflower Stalk: Application of Chemical and Biological Pretreatments by Response Surface Methodology (RSM). Biomass Convers. Biorefinery 2021, 11 (5), 1759–1773. 10.1007/s13399-020-00602-7.

[ref11] Karmoker J. R.; Hasan I.; Ahmed N.; Saifuddin M.; Reza M. S. Development and Optimization of Acyclovir Loaded Mucoadhesive Microspheres by Box – Behnken Design. Dhaka Univ. J. Pharm. Sci. 2019, 18, 1–12. 10.3329/dujps.v18i1.41421.

[ref12] Ahmed N.; Mir F. Q. Box–Behnken Design for Optimization of Iron Removal by Hybrid Oxidation–Microfiltration Process Using Ceramic Membrane. J. Mater. Sci. 2022, 57 (32), 15224–15238. 10.1007/s10853-022-07567-0.

[ref13] Bhattacharya S. Central Composite Design for Response Surface Methodology and Its Application in Pharmacy; Kayaroganam P., Ed.; IntechOpen: Rijeka, 2021; Ch. 5.

[ref14] Kumar A.; Shende D.; Wasewar K. Central Composite Design Approach for Optimization of Levulinic Acid Separation by Reactive Components. Ind. Eng. Chem. Res. 2021, 60 (37), 13692–13700. 10.1021/acs.iecr.1c02589.

[ref15] Khuri A. I.; Mukhopadhyay S. Response Surface Methodology. WIREs Comput. Stat. 2010, 2 (2), 128–149. 10.1002/wics.73.

[ref16] Jones B. Definitive Screening Designs with Added Two-Level Factors. J. Qual. Technol. 2013, 45, 120.

[ref17] Takagaki K.; Ito T.; Arai H.; Obata Y.; Takayama K.; Onuki Y. The Usefulness of Definitive Screening Design for a Quality by Design Approach as Demonstrated by a Pharmaceutical Study of Orally Disintegrating Tablet. Chem. Pharm. Bull. 2019, 67 (10), 1144–1151. 10.1248/cpb.c19-00553. 31582634

[ref18] Greenhill S.; Rana S.; Gupta S.; Vellanki P.; Venkatesh S. Bayesian Optimization for Adaptive Experimental Design: A Review. IEEE Access 2020, 8, 13937–13948. 10.1109/ACCESS.2020.2966228.

[ref19] Shields B. J.; Stevens J.; Li J.; Parasram M.; Damani F.; Alvarado J. I. M.; Janey J. M.; Adams R. P.; Doyle A. G. Bayesian Reaction Optimization as a Tool for Chemical Synthesis. Nature 2021, 590 (7844), 89–96. 10.1038/s41586-021-03213-y. 33536653

[ref20] Torres J. A. G.; Lau S. H.; Anchuri P.; Stevens J. M.; Tabora J. E.; Li J.; Borovika A.; Adams R. P.; Doyle A. G. A Multi-Objective Active Learning Platform and Web App for Reaction Optimization. J. Am. Chem. Soc. 2022, 144 (43), 19999–20007. 10.1021/jacs.2c08592. 36260788

[ref21] Snoek J.; Larochelle H.; Adams R. P. Practical Bayesian Optimization of Machine Learning Algorithms. Adv. Neural Inf. Process. Syst. 2012, 25.

[ref22] Schulz E.; Speekenbrink M.; Krause A. A Tutorial on Gaussian Process Regression: Modelling, Exploring, and Exploiting Functions. J. Math. Psychol. 2018, 85, 1–16. 10.1016/j.jmp.2018.03.001.

[ref23] Deringer V. L.; Bartók A. P.; Bernstein N.; Wilkins D. M.; Ceriotti M.; Csányi G. Gaussian Process Regression for Materials and Molecules. Chem. Rev. 2021, 121 (16), 10073–10141. 10.1021/acs.chemrev.1c00022. 34398616 PMC8391963

[ref24] Schweidtmann A. M.; Clayton A. D.; Holmes N.; Bradford E.; Bourne R. A.; Lapkin A. A. Machine Learning Meets Continuous Flow Chemistry: Automated Optimization towards the Pareto Front of Multiple Objectives. Chem. Eng. J. 2018, 352, 277–282. 10.1016/j.cej.2018.07.031.

[ref25] Kondo M.; Wathsala H. D. P.; Sako M.; Hanatani Y.; Ishikawa K.; Hara S.; Takaai T.; Washio T.; Takizawa S.; Sasai H. Exploration of Flow Reaction Conditions Using Machine-Learning for Enantioselective Organocatalyzed Rauhut–Currier and [3 + 2] Annulation Sequence. Chem. Commun. 2020, 56 (8), 1259–1262. 10.1039/C9CC08526B. 31903462

[ref26] Griffiths R.-R.; Hernández-Lobato J. M. Constrained Bayesian Optimization for Automatic Chemical Design Using Variational Autoencoders. Chem. Sci. 2020, 11 (2), 577–586. 10.1039/C9SC04026A. 32190274 PMC7067240

[ref27] Cho H.; Kim Y.; Lee E.; Choi D.; Lee Y.; Rhee W. Basic Enhancement Strategies When Using Bayesian Optimization for Hyperparameter Tuning of Deep Neural Networks. IEEE Access 2020, 8, 52588–52608. 10.1109/ACCESS.2020.2981072.

[ref28] Wu J.; Chen X.-Y.; Zhang H.; Xiong L.-D.; Lei H.; Deng S.-H. Hyperparameter Optimization for Machine Learning Models Based on Bayesian Optimizationb. J. Electron. Sci. Technol. 2019, 17 (1), 26–40.

[ref29] Duris J.; Kennedy D.; Hanuka A.; Shtalenkova J.; Edelen A.; Baxevanis P.; Egger A.; Cope T.; McIntire M.; Ermon S.; Ratner D. Bayesian Optimization of a Free-Electron Laser. Phys. Rev. Lett. 2020, 124 (12), 124801 10.1103/PhysRevLett.124.124801. 32281869

[ref30] Sakurai A.; Yada K.; Simomura T.; Ju S.; Kashiwagi M.; Okada H.; Nagao T.; Tsuda K.; Shiomi J. Ultranarrow-Band Wavelength-Selective Thermal Emission with Aperiodic Multilayered Metamaterials Designed by Bayesian Optimization. ACS Cent. Sci. 2019, 5 (2), 319–326. 10.1021/acscentsci.8b00802. 30834320 PMC6396383

[ref31] Tallorin L.; Wang J.; Kim W. E.; Sahu S.; Kosa N. M.; Yang P.; Thompson M.; Gilson M. K.; Frazier P. I.; Burkart M. D.; Gianneschi N. C. Discovering de Novo Peptide Substrates for Enzymes Using Machine Learning. Nat. Commun. 2018, 9 (1), 5253 10.1038/s41467-018-07717-6. 30531862 PMC6286390

[ref32] Rosa S. S.; Nunes D.; Antunes L.; Prazeres D. M. F.; Marques M. P. C.; Azevedo A. M. Maximizing MRNA Vaccine Production with Bayesian Optimization. Biotechnol. Bioeng. 2022, 119 (11), 3127–3139. 10.1002/bit.28216. 36017534 PMC9539360

[ref33] Jang W. D.; Kim G. B.; Kim Y.; Lee S. Y. Applications of Artificial Intelligence to Enzyme and Pathway Design for Metabolic Engineering. Curr. Opin. Biotechnol. 2022, 73, 101–107. 10.1016/j.copbio.2021.07.024. 34358728

[ref34] Bedbrook C. N.; Yang K. K.; Robinson J. E.; Mackey E. D.; Gradinaru V.; Arnold F. H. Machine Learning-Guided Channelrhodopsin Engineering Enables Minimally Invasive Optogenetics. Nat. Methods 2019, 16 (11), 1176–1184. 10.1038/s41592-019-0583-8. 31611694 PMC6858556

[ref35] Wittmann B. J.; Johnston K. E.; Wu Z.; Arnold F. H. Advances in Machine Learning for Directed Evolution. Curr. Opin. Struct. Biol. 2021, 69, 11–18. 10.1016/j.sbi.2021.01.008. 33647531

[ref36] Pandi A.; Diehl C.; Yazdizadeh Kharrazi A.; Scholz S. A.; Bobkova E.; Faure L.; Nattermann M.; Adam D.; Chapin N.; Foroughijabbari Y.; Moritz C.; Paczia N.; Cortina N. S.; Faulon J.-L.; Erb T. J. A Versatile Active Learning Workflow for Optimization of Genetic and Metabolic Networks. Nat. Commun. 2022, 13 (1), 3876 10.1038/s41467-022-31245-z. 35790733 PMC9256728

[ref37] Ginsbourger D.; Le Riche R.; Carraro L. Kriging Is Well-Suited to Parallelize Optimization BT - Computational Intelligence in Expensive Optimization Problems; Tenne Y., Goh C.-K., Eds.; Springer: Berlin, Heidelberg, 2010; pp 131–162.

[ref38] Müller M.; Sprenger G. A.; Pohl M. CC Bond Formation Using ThDP-Dependent Lyases. Curr. Opin. Chem. Biol. 2013, 17 (2), 261–270. 10.1016/j.cbpa.2013.02.017. 23523314

[ref39] Iding H.; Dünnwald T.; Greiner L.; Liese A.; Müller M.; Siegert P.; Grötzinger J.; Demir A. S.; Pohl M. Benzoylformate Decarboxylase from Pseudomonas Putida as Stable Catalyst for the Synthesis of Chiral 2-Hydroxy Ketones. Chem. – A Eur. J. 2000, 6 (8), 1483–1495. 10.1002/(SICI)1521-3765(20000417)6:8<1483::AID-CHEM1483>3.0.CO;2-S. 10840971

[ref40] Demir A. S.; Dünnwald T.; Iding H.; Pohl M.; Müller M. Asymmetric Benzoin Reaction Catalyzed by Benzoylformate Decarboxylase. Tetrahedron: Asymmetry 1999, 10 (24), 4769–4774. 10.1016/S0957-4166(99)00516-9.

[ref41] Parmeggiani F.; Lovelock S. L.; Weise N. J.; Ahmed S. T.; Turner N. J. Synthesis of D- and L-Phenylalanine Derivatives by Phenylalanine Ammonia Lyases: A Multienzymatic Cascade Process. Angew. Chemie Int. Ed. 2015, 54 (15), 4608–4611. 10.1002/anie.201410670. PMC4531825 25728350

[ref42] Lovelock S. L.; Lloyd R. C.; Turner N. J. Phenylalanine Ammonia Lyase Catalyzed Synthesis of Amino Acids by an MIO-Cofactor Independent Pathway. Angew. Chemie Int. Ed. 2014, 53 (18), 4652–4656. 10.1002/anie.201311061. 24692092

[ref43] Cui J. D.; Qiu J. Q.; Fan X. W.; Jia S. R.; Tan Z. L. Biotechnological Production and Applications of Microbial Phenylalanine Ammonia Lyase: A Recent Review. Crit. Rev. Biotechnol. 2014, 34 (3), 258–268. 10.3109/07388551.2013.791660. 23688066

[ref44] Arafa A.; Abdel-Ghani A.; El-Dahmy S.; Abdelaziz S.; El-Ayouty Y.; El-Sayed A. Purification and Characterization of Anabaena Flos-Aquae Phenylalanine Ammonia-Lyase as a Novel Approach for Myristicin Biotransformation. J. Microbiol. Biotechnol. 2020, 30, 622–632. 31581382 10.4014/jmb.1908.08009 PMC9728195

[ref45] Sarkissian C. N.; Kang T. S.; Gámez A.; Scriver C. R.; Stevens R. C. Evaluation of Orally Administered PEGylated Phenylalanine Ammonia Lyase in Mice for the Treatment of Phenylketonuria. Mol. Genet. Metab. 2011, 104 (3), 249–254. 10.1016/j.ymgme.2011.06.016. 21803624 PMC3205297

[ref46] González B.; Vicuña R. Benzaldehyde Lyase, a Novel Thiamine PPi-Requiring Enzyme, from Pseudomonas Fluorescens Biovar I. J. Bacteriol. 1989, 171 (5), 2401–2405. 10.1128/jb.171.5.2401-2405.1989. 2496105 PMC209914

[ref47] Shahriari B.; Swersky K.; Wang Z.; Adams R. P.; de Freitas N. Taking the Human Out of the Loop: A Review of Bayesian Optimization. Proc. IEEE 2016, 104 (1), 148–175. 10.1109/JPROC.2015.2494218.

[ref48] Archetti F.; Candelieri A. Bayesian Optimization and Data Science; Springer, 2019.

[ref49] Zhan D.; Xing H. Expected Improvement for Expensive Optimization: A Review. J. Glob. Optim. 2020, 78 (3), 507–544. 10.1007/s10898-020-00923-x.

[ref50] Zhang L.; Jin G.; Liu T.; Zhang R. Generalized Hierarchical Expected Improvement Method Based on Black-Box Functions of Adaptive Search Strategy. Appl. Math. Model. 2022, 106, 30–44. 10.1016/j.apm.2021.12.041.

[ref51] Häse F.; Aldeghi M.; Hickman R. J.; Roch L. M.; Aspuru-Guzik A. Gryffin: An Algorithm for Bayesian Optimization of Categorical Variables Informed by Expert Knowledge. Appl. Phys. Rev. 2021, 8 (3), 031406 10.1063/5.0048164.

[ref52] Christensen M.; Yunker L. P. E.; Adedeji F.; Häse F.; Roch L. M.; Gensch T.; dos Passos Gomes G.; Zepel T.; Sigman M. S.; Aspuru-Guzik A.; Hein J. E. Data-Science Driven Autonomous Process Optimization. Commun. Chem. 2021, 4 (1), 112 10.1038/s42004-021-00550-x. 36697524 PMC9814253

[ref53] Xu J.; Grosslight S.; Mack K. A.; Nguyen S. C.; Clagg K.; Lim N.-K.; Timmerman J. C.; Shen J.; White N. A.; Sirois L. E.; Han C.; Zhang H.; Sigman M. S.; Gosselin F. Atroposelective Negishi Coupling Optimization Guided by Multivariate Linear Regression Analysis: Asymmetric Synthesis of KRAS G12C Covalent Inhibitor GDC-6036. J. Am. Chem. Soc. 2022, 144 (45), 20955–20963. 10.1021/jacs.2c09917. 36326518

[ref54] Wang Y.; Chen T.-Y.; Vlachos D. G. NEXTorch: A Design and Bayesian Optimization Toolkit for Chemical Sciences and Engineering. J. Chem. Inf. Model. 2021, 61 (11), 5312–5319. 10.1021/acs.jcim.1c00637. 34694805

[ref55] Müller C. R.; Pérez-Sánchez M.; Domínguez de María P. Benzaldehyde Lyase-Catalyzed Diastereoselective C–C Bond Formation by Simultaneous Carboligation and Kinetic Resolution. Org. Biomol. Chem. 2013, 11 (12), 2000–2004. 10.1039/c2ob27344f. 23280121

[ref56] Gardner J. R.; Kusner M. J.; Xu Z. E.; Weinberger K. Q.; Cunningham J. P. Bayesian Optimization with Inequality Constraints. In Proceedings of the 31st International Conference on Machine Learning; JMLR, 2014; Vol. 32; pp 937–945.

[ref57] Bradford E.; Schweidtmann A. M.; Lapkin A. Efficient Multiobjective Optimization Employing Gaussian Processes, Spectral Sampling and a Genetic Algorithm. J. Glob. Optim. 2018, 71 (2), 407–438. 10.1007/s10898-018-0609-2.

[ref58] Knowles J. ParEGO: A Hybrid Algorithm with on-Line Landscape Approximation for Expensive Multiobjective Optimization Problems. IEEE Trans. Evol. Comput. 2006, 10 (1), 50–66. 10.1109/TEVC.2005.851274.

