# Supplementary figures and images for: A Customized Bayesian Algorithm to Optimize Enzyme-Catalyzed Reactions
Source: ACS Sustain Chem Eng. 2023 Aug 3;11(33):12336–44. doi: 10.1021/acssuschemeng.3c02402 (PMC10445256; doi:10.1021/acssuschemeng.3c02402)

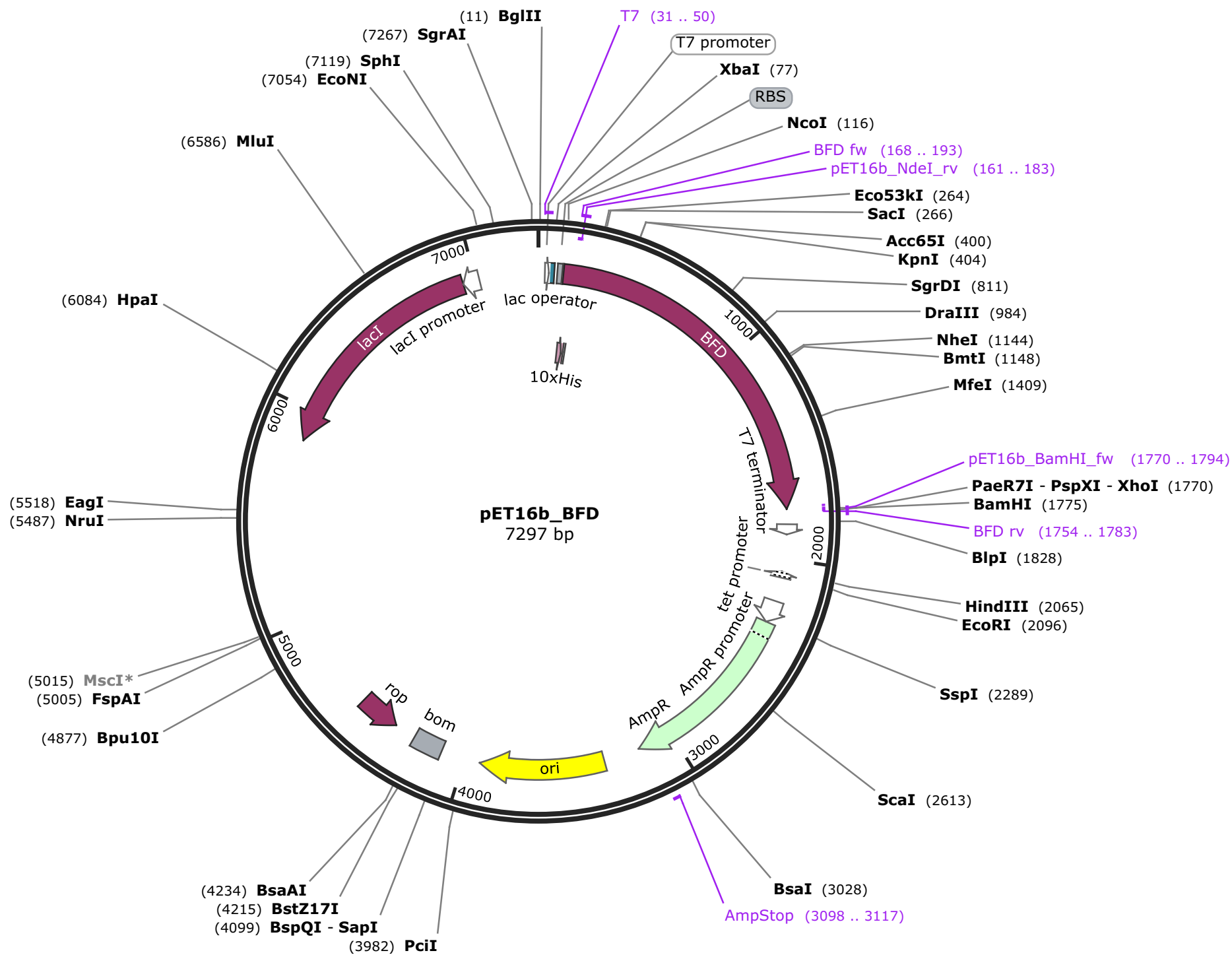

Supplement: Supplementary file 1 — sc3c02402_si_001.pdf [file sc3c02402_si_001.pdf]

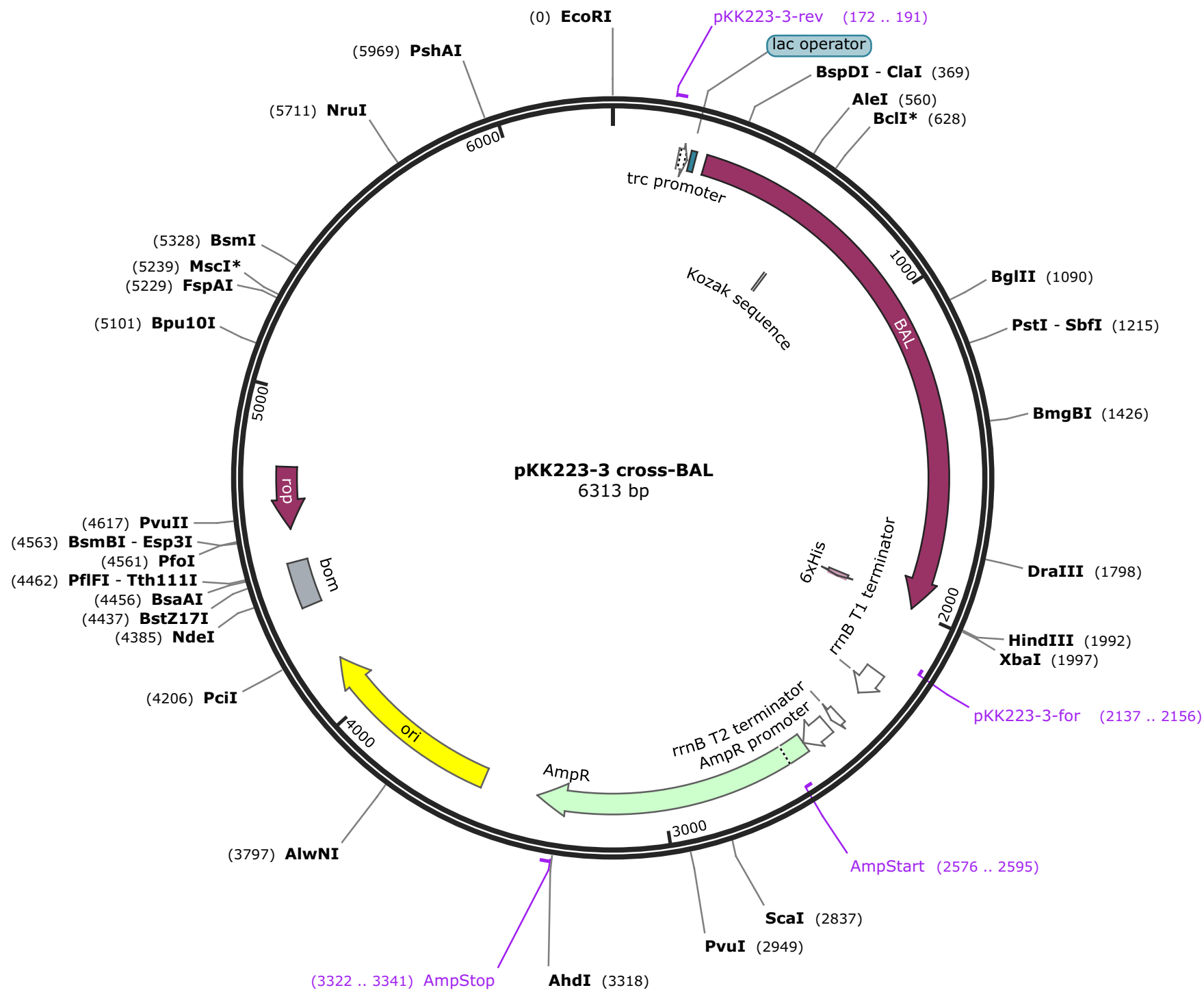

Supplement: Supplementary file 2 — sc3c02402_si_002.pdf [file sc3c02402_si_002.pdf]

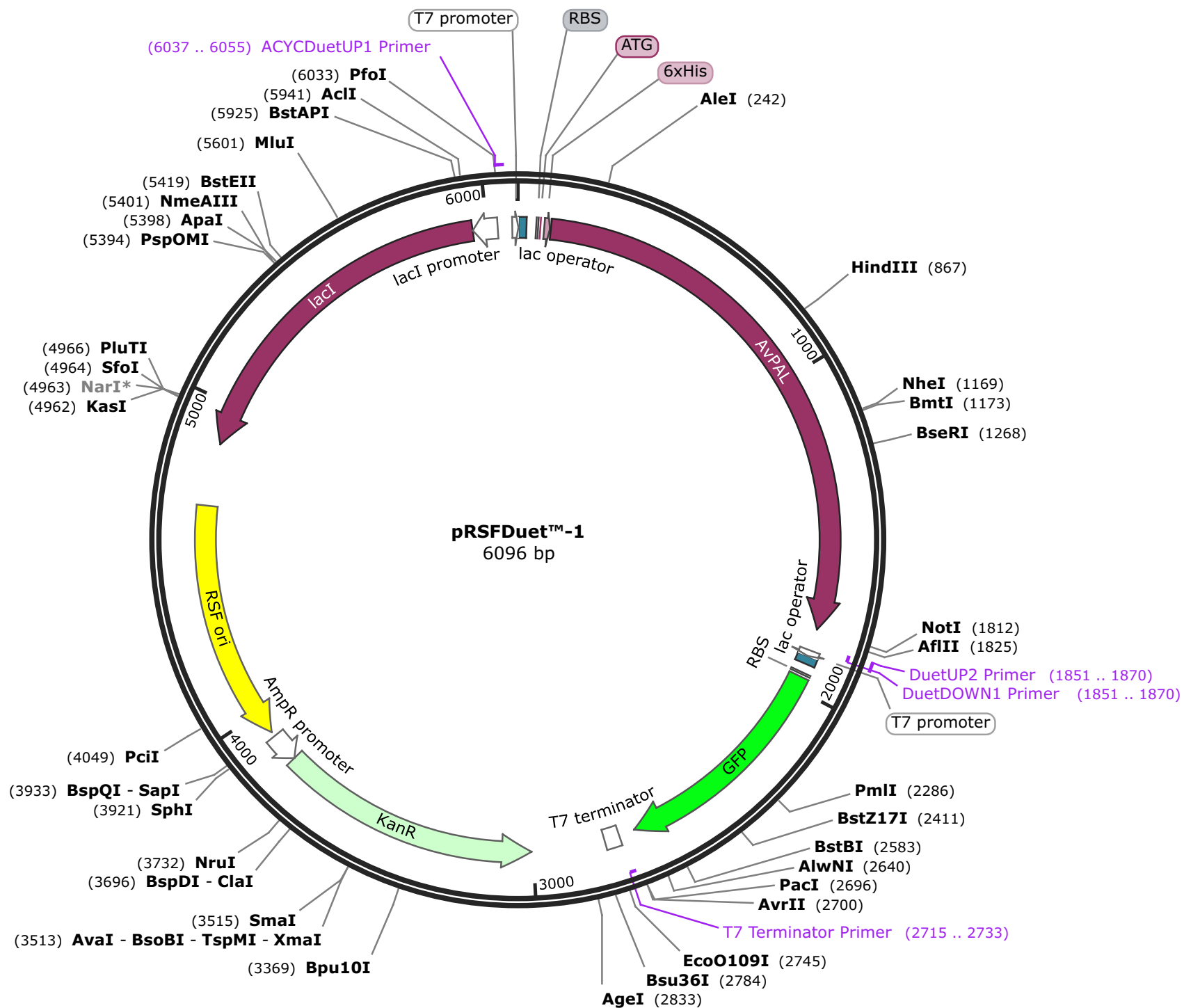

Supplement: Supplementary file 3 — sc3c02402_si_003.pdf [file sc3c02402_si_003.pdf]
