# Supplementary material for: A Customized Bayesian Algorithm to Optimize Enzyme-Catalyzed Reactions
Source: ACS Sustain Chem Eng. 2023 Aug 3;11(33):12336–44. doi: 10.1021/acssuschemeng.3c02402 (PMC10445256; doi:10.1021/acssuschemeng.3c02402)
Supplement: Supplementary file 4 — sc3c02402_si_004.pdf [file sc3c02402_si_004.pdf]

Supplementary Information

A customized Bayesian algorithm to optimize enzyme-catalyzed reactions

Authors:
Ryo Tachibana,* Kailin Zhang, Zhi Zou, Simon Burgener and Thomas R. Ward*

Table of contents

Supplementary figures S2
Material and Method S15
  General information and instrumentation S15
  Expression and purification of enzymes S15
  Experimental procedure S17
Generalized algorithm of BOA S21
Availability of the program S21
Synthesis S22
Supplementary References S25

Supplementary figures

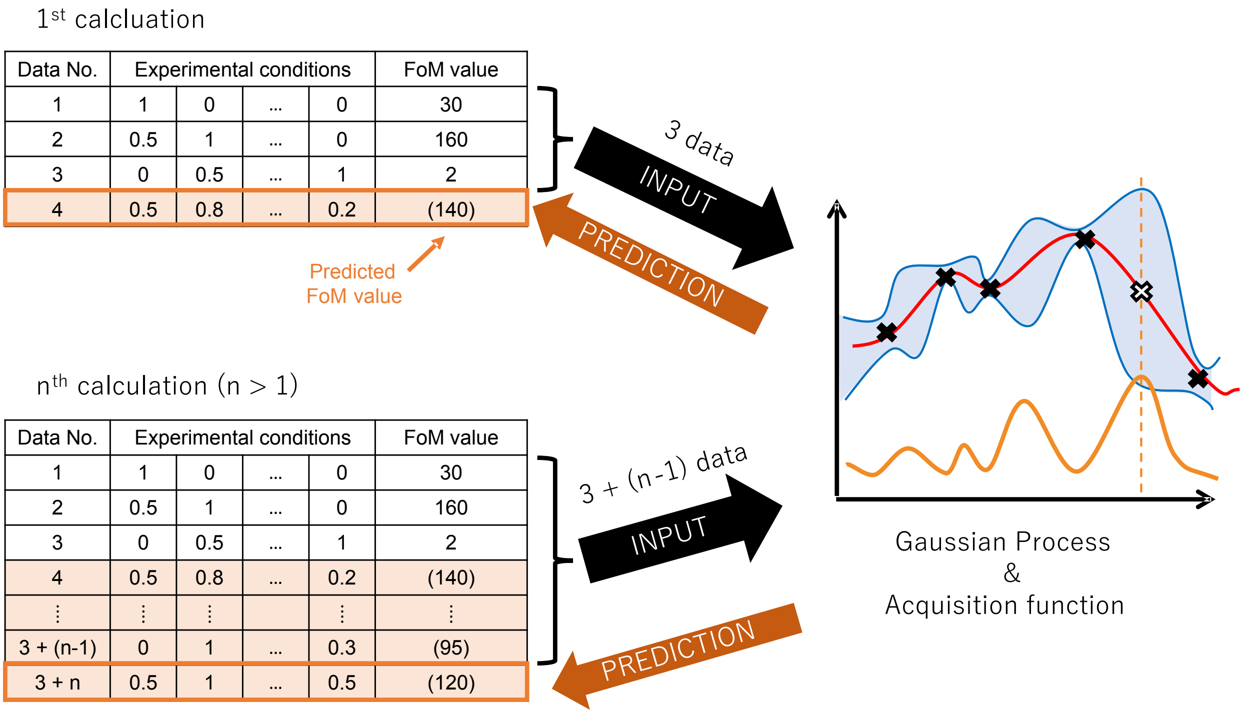

Figure S 1 Overview of the Kriging believer algorithm. In identifying the second and subsequent conditions to be tested, it is assumed that the predictions of the previously suggested conditions and the FoM value are correct, and these are temporarily added to the data set.

No.	Substrate (mM)	Enzyme (μM)	pH	TPP (mM)	DMSO (%)	TON
**1**	50.5	10.5	7	0.5	50	**303**
**2**	1	1	8	0	10	**0**
**3**	100	1	6	1	50	**783**
**4**	100	1	8	1	10	**2749**
**5**	1	1	6	0	50	**0**
**6**	1	1	8	1	50	**0**
**7**	50.5	10.5	7	1	30	**513**
**8**	100	20	6	1	10	**742**
**9**	100	10.5	7	0.5	30	**1157**
**10**	50.5	10.5	7	0.5	30	**487**
**11**	1	20	6	0	10	**1**
**12**	1	20	6	1	50	**2**
**13**	100	20	8	0	10	**64**
**14**	1	20	8	1	10	**1**
**15**	1	1	6	1	10	**10**
**16**	50.5	10.5	7	0.5	30	**496**
**17**	50.5	10.5	7	0	30	**60**
**18**	1	20	8	0	50	**0**
**19**	100	1	8	0	50	**38**
**20**	100	1	6	0	10	**362**
**21**	50.5	10.5	7	0.5	10	**585**
**22**	100	20	6	0	50	**113**
**23**	50.5	10.5	6	0.5	30	**574**
**24**	50.5	10.5	8	0.5	30	**457**
**25**	50.5	1	7	0.5	30	**975**
**26**	100	20	8	1	50	**470**
**27**	50.5	20	7	0.5	30	**300**
**28**	1	10.5	7	0.5	30	**3**
**29**	50.5	10.5	7	0.5	30	**553**

Table S 1 The experimental table for the BFD reaction generated by MODDE® and the resulting TONs.

No.	Substrate (mM)	Enzyme (µM)	pH	NH_4^+ (M)	DMSO (%)	TON
**1**	50	1	11	1.73	5	**500**
**2**	50	20	11	5	5	**186**
**3**	25.05	10.5	9.5	2.59	25.50	**561**
**4**	50	20	8	4.01	21.67	**766**
**5**	50	1	10	0.1	5	**76**
**6**	0.1	20	8	5	5	**3**
**7**	0.1	1	8	0.1	5	**0**
**8**	50	13.67	8	5	5	**1531**
**9**	50	20	8	0.1	5	**0**
**10**	50	1	9	5	27.45	**2440**
**11**	0.1	20	11	2.03	50	**0**
**12**	0.1	1	11	0.1	50	**0**
**13**	16.73	1	11	5	27.45	**178**
**14**	25.05	10.5	9.5	2.59	25.50	**537**
**15**	50	20	11	0.1	50	**3**
**16**	0.1	1	11	5	5	**55**
**17**	33.37	1	8	5	5	**2252**
**18**	0.1	1	8	3.42	50	**0**
**19**	33.37	1	11	0.1	5	**220**
**20**	25.05	10.5	9.5	2.55	5	**724**
**21**	50	20	8	2.03	50	**2**
**22**	25.05	10.5	9.5	2.59	25.50	**547**
**23**	0.1	1	8	0.1	35	**0**
**24**	0.1	20	8	0.1	50	**0**
**25**	0.1	13.67	11	5	14.15	**4**
**26**	50	7.33	11	0.1	5	**94**
**27**	0.1	20	11	0.1	5	**0**
**28**	50	1	8	0.1	50	**0**
**29**	50	1	11	3.42	50	**362**

Table S 2 Experimental table for the PAL reaction generated by MODDE® and the resulting TONs.

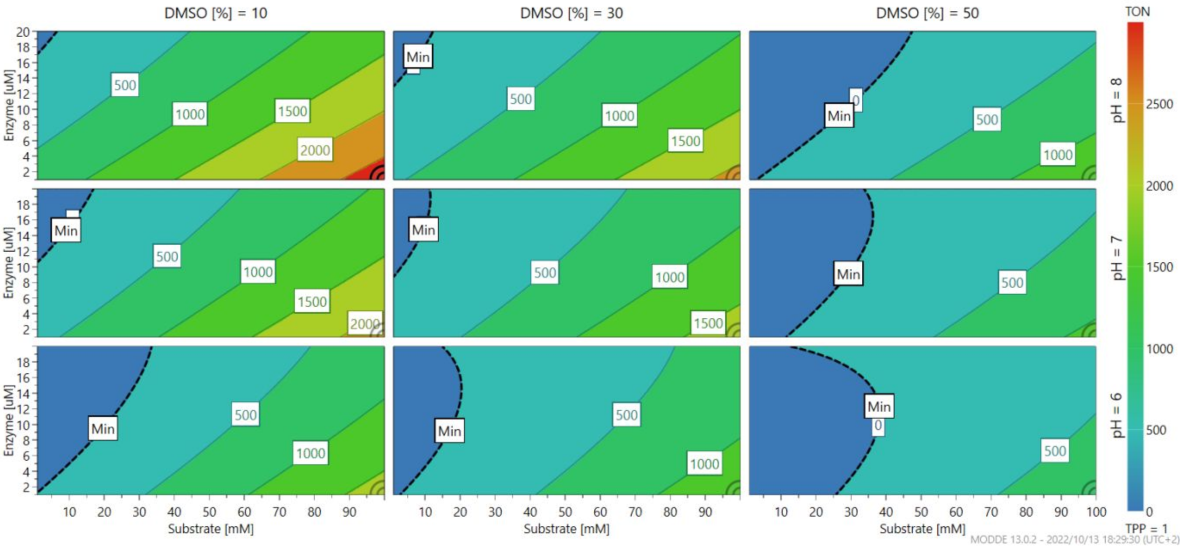

Figure S 2 Contour plot for the BFD reaction with TPP = 1 mM. TONs are represented by different colors. Variables include substrate concentration, enzyme concentration, % DMSO and pH. Graph generated by MODDE®.

Factor	Value	Unit
Substrate	100	mM
Enzyme	1	uM
pH	8	
TPP	1	mM
DMSO	10	%
Predicted TON	2776	
Measured TON	3289	

Table S 3 Best condition predicted by MODDE® and corresponding TONs determined for the BFD reaction.

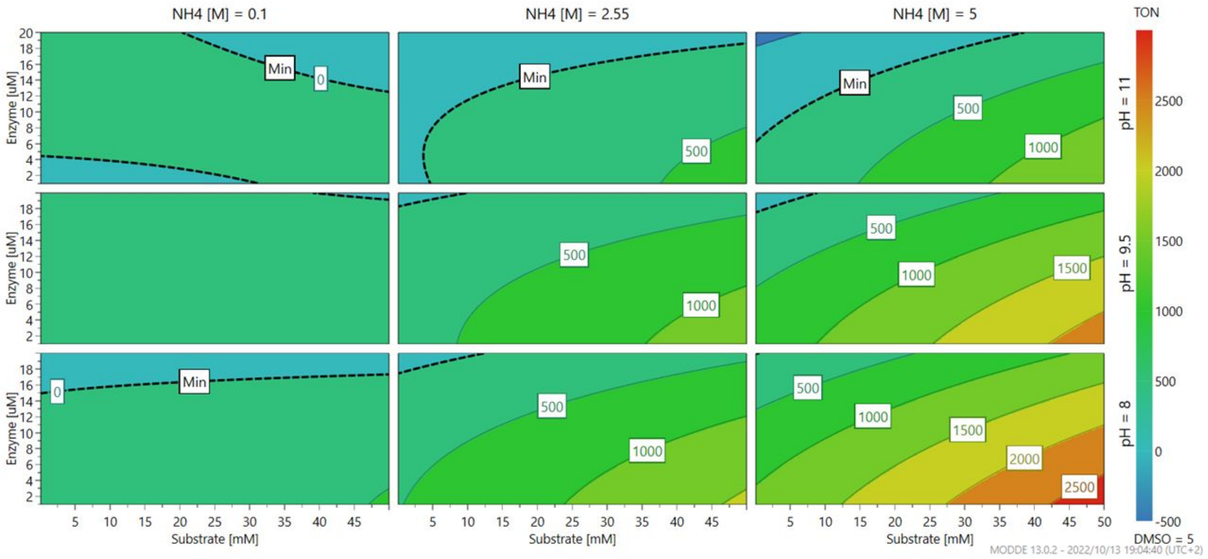

Figure S 3 Contour plot for the PAL reaction with DMSO = 5%. TONs are represented by different colors. Variables include substrate concentration, enzyme concentration, NH_4^+ concentration and pH. Graph generated by MODDE®.

Factor	Value	Unit
Substrate	100	mM
Enzyme	1	uM
pH	8	
NH_4^+	5	M
DMSO	5	%
Predicted TON	2764	
Measured TON	1544	

Table S 4 Best condition predicted by MODDE® and corresponding TONs determined for the PAL reaction.

Cycle	Substrate (mM)	Enzyme (μM)	pH	TPP (mM)	DMSO (%)	Predicted TON	Measured TON
Initial Data	100	1	6	0	10	-	362
	100	20	8	1	50	-	470
	50.5	10.5	6	0.5	30	-	574
	50.5	20	7	0.5	30	-	300
	1	1	8	1	50	-	0
Cycle 1	100	20	6	0.7	38	417	816
	100	18.1	6	1	18	411	1377
	100	18.1	6	0.1	50	411	669
	100	20	6	1	50	415	566
	100	18.1	6	0.4	26	420	1152
Cycle 2	100	20	6	1	10	932	1424
	100	20	6	0.9	10	932	1417
	100	18.1	6	1	10	931	1573
	100	20	6	1	14	933	1462
	100	20	6	0.8	10	931	1363
Cycle 3	90.1	20	6	1	10	1285	1089
	100	20	6.2	1	10	1286	1169
	100	16.2	6	1	10	1268	1304
	90.1	18.1	6	1	10	1270	1139
	100	18.1	6	0.9	10	1272	1251
Cycle 4	100	18.1	6.2	1	10	1235	1218
	90.1	20	6	0.9	10	1234	989
	100	20	6.2	0.9	10	1234	1072
	80.2	20	6	1	10	1228	920
	90.1	20	6.2	1	10	1230	1024
RSM	100	1	8	1	10		3289

Table S 5 Experimental table for the BFD reaction based on the conventional Expected Improvement function. The best result generated with the response surface method (RSM) is listed for comparison.

Cycle	Substrate (mM)	Enzyme (μM)	pH	NH_4^+ (M)	DMSO (%)	Predicted TON	Measured TON
Initial Data	0.1	1	11	0.1	50	-	0
	0.1	20	8	5	5	-	3
	25.1	10.5	9.5	2.6	25.5	-	537
	50	20	11	5	5	-	186
	50	1	8	0.1	50	-	0
Cycle 1	40.02	10.5	10.4	2.55	9.5	245	212
	40.02	12.4	10.4	4.02	27.5	246	167
	40.02	20	10.4	0.59	14	236	157
	40.02	1	10.4	4.51	5	236	157
	40.02	16.2	10.4	3.53	32	246	169
Cycle 2	25.05	8.6	9.5	1.57	5	204	578
	30.04	8.6	9.5	2.06	5	205	705
	25.05	10.5	9.8	4.02	23	205	577
	30.04	10.5	9.8	2.06	5	207	519
	30.04	12.4	9.8	2.55	14	208	497
Cycle 3	10.08	4.8	8.6	0.59	5	443	200
	15.07	4.8	8.6	1.08	5	448	457
	15.07	4.8	8.6	0.59	5	448	329
	15.07	4.8	8.9	1.08	5	451	501
	10.08	4.8	8.9	1.08	5	450	351
RSM	50	1	9	5	27.45		2440

Table S 6 Experimental table for the PAL reaction based on the conventional Expected Improvement function. The best result generated with the response surface method (RSM) is listed for comparison.

Cycle	Substrate (mM)	Enzyme (µM)	pH	TPP (mM)	DMSO (%)	Predicted TON	Measured TON
Initial Data	100	1	6	0	10	-	362
	100	20	8	1	50	-	470
	50.5	10.5	6	0.5	30	-	574
	50.5	20	7	0.5	30	-	300
	1	1	8	1	50	-	0
Cycle 1	100	20	6	0.7	38	417	907
	100	6.7	6	1	10	398	2377
	100	6.7	6	0	50	400	163
	60.4	20	6	0	14	404	250
	20.8	20	6	1	10	392	182
Cycle 2	100	2.9	6	1	10	1194	3068
	100	1	8	1	10	861	3189
	100	20	7	1	10	872	1163
	100	1	6	1	50	823	727
	1	1	6	1	10	743	0
Cycle 3	100	1	7.6	1	10	2040	4020
	100	12.4	6	1	14	1529	1732
	100	1	8	0	10	1264	71
	100	20	8	1	10	1337	1187
	100	1	8	1	50	1129	265
Cycle 4	100	1	7.4	1	10	2336	4457
	100	16.2	6.2	1	10	1551	1488
	1	1	8	1	10	1199	7
	100	1	6	1	42	1245	1010
	50.5	18.1	8	1	10	1152	526
Cycle 5	100	1	7.2	1	10	2605	4849
	100	16.2	6	1	14	1495	1495
	100	18.1	8	0.8	10	1353	1296
	100	2.9	6	0.2	10	1184	3721
	100	1	6	0.9	42	1215	1101
RSM	100	1	8	1	10		3289

Table S 7 Optimization process of the BFD reaction using BOA-1. The best result generated with the response surface method (RSM) is listed for comparison.

Cycle	Substrate (mM)	Enzyme (μM)	pH	NH_4^+ (M)	DMSO (%)	Predicted TON	Measured TON
Initial Data	0.1	1	11	0.1	50	-	0
	0.1	20	8	5	5	-	3
	25.1	10.5	9.5	2.6	25.5	-	537
	50	20	11	5	5	-	186
	50	1	8	0.1	50	-	0
Cycle 1	40.02	10.5	10.4	2.55	9.5	245	288
	50	20	11	1.57	45.5	215	144
	45.01	1	8.3	5	9.5	212	2726
	5.09	1	11	5	5	212	81
	5.09	20	10.7	0.1	5	213	3
Cycle 2	50	1	8	5	5	1301	2454
	0.1	1	8	5	9.5	790	91
	45.01	1	8	0.1	5	758	0
	50	1	11	5	9.5	769	51
	30.04	1	8	3.53	45.5	763	603
Cycle 3	50	1	8	5	9.5	1439	2584
	50	20	8	5	5	893	1086
	50	4.8	8	3.04	50	711	386
	0.1	1	8	5	5	632	65
	50	1	11	5	5	643	12
Cycle 4	50	2.9	8	5	5	1602	2322
	50	20	8	3.53	23	818	731
	50	1	8	3.04	50	740	193
	50	1	8	0.1	5	665	0
	50	1	10.7	4.51	32	513	178
Cycle 5	50	4.8	8	5	5	1640	2018
	50	20	8	2.06	9.5	678	477
	50	1	8	3.04	45.5	681	191
	5.09	1	8	5	23	603	1056
	50	18.1	10.4	5	5	552	193
RSM	50	1	9	5	27.45		2440

Table S 8 Optimization process of the PAL reaction using BOA-1. The best result generated with the response surface method (RSM) is listed for comparison.

Cycle	Substrate (mM)	Enzyme (μM)	pH	TPP (mM)	DMSO (%)	Predicted TON	Measured TON
Initial Data	100	1	6	0	10	-	362
	100	20	8	1	50	-	470
	50.5	10.5	6	0.5	30	-	574
	50.5	20	7	0.5	30	-	300
	1	1	8	1	50	-	0
Cycle 1	100	20	6	1	10	400	1255
	100	20	6	0	50	404	144
	1	20	6	0	10	387	1
	100	1	6	1	50	378	808
	100	20	8	0	10	369	55
Cycle 2	100	1	6	1	10	661	4210
	100	1	8	1	10	465	3100
	100	20	6	1	14	753	1198
	1	1	6	1	10	484	0
	100	20	6	1	50	622	440
Cycle 3	100	1	7.8	1	10	2066	3828
	100	1	6.2	1	10	2125	5684
	100	1	8	0	50	744	17
	100	1	7.6	1	10	2114	4023
	100	1	6.4	1	10	2153	5888
Cycle 4	1	1	8	0	10	945	0
	1	20	8	1	10	1033	0
	100	1	6.6	1	10	3685	5910
	100	1	6.8	1	10	3678	5645
	100	1	7	1	10	3648	3940
Cycle 5	1	1	6	0	50	442	0
	1	20	8	0	50	323	0
	100	1	6	1	14	3977	4032
	100	1	7.2	1	10	4121	4955
	100	1	6	0.9	10	3971	4170
RSM	100	1	8	1	10		3289

Table S 9 Optimization process of the BDF reaction using BOA-2. The best result generated with the response surface method (RSM) is listed for comparison.

Cycle	Substrate (mM)	Enzyme (μM)	pH	NH_4^+ (M)	DMSO (%)	Predicted TON	Measured TON
Initial Data	0.1	1	11	0.1	50	-	0
	0.1	20	8	5	5	-	3
	25.1	10.5	9.5	2.6	25.5	-	537
	50	20	11	5	5	-	186
	50	1	8	0.1	50	-	0
Cycle 1	50	1	11	0.1	5	196	211
	50	20	11	0.1	50	192	5
	0.1	1	11	5	5	200	0
	50	1	8	5	5	202	2477
	50	20	8	0.1	5	202	0
Cycle 2	50	1	8	5	9.5	1173	2621
	0.1	1	8	0.1	5	406	0
	45.01	1	8	5	5	1175	2765
	50	1	8	5	23	1097	1575
	0.1	1	8	4.02	41	521	61
Cycle 3	50	1	11	5	23	930	6
	50	20	8	5	5	1002	1053
	50	1	8.3	5	5	1836	3174
	0.1	20	8	0.1	50	174	0
	50	2.9	8	5	5	1834	2608
Cycle 4	0.1	20	11	0.1	5	135	0
	50	1	8	4.51	5	2085	2581
	50	1	8	0.1	5	1035	0
	40.02	1	8	5	5	2009	2859
	50	4.8	8	5	5	2034	2407
Cycle 5	0.1	1	8	5	5	1185	84
	50	1	8	5	14	2147	1873
	50	1	8.6	5	5	2119	3260
	45.01	2.9	8	5	5	2175	2338
	50	1	8	5	18.5	2057	1727
RSM	50	1	9	5	27.45		2440

Table S 10 Optimization process of PAL reactions by BOA-2 and comparison with the best result by response surface method (RSM).

No.	Benzaldehyde (mM)	Isobutyraldehyde (mM)	Enzyme (µM)	pH	TPP (mM)	DMSO (%)	Total TON	Chemoselectivity
1	100	1	20	8	1	10	27	9.5%
2	50.5	100.5	10.5	7	0.5	10	1865	3.2%
3	1	100.5	10.5	7	0.5	30	85	5.7%
4	100	200	20	6	0	10	6	100%
5	1	200	20	8	0	50	5	45.4%
6	1	200	1	8	1	50	726	33.0%
7	1	1	20	8	1	50	4	13.5%
8	1	1	1	6	0	50	0	0.0%
9	100	1	1	6	0	10	0	0.0%
10	100	200	20	6	1	50	156	95.1%
11	100	1	20	6	1	10	7	14.0%
12	50.5	100.5	10.5	7	0.5	30	2465	0.6%
13	100	200	1	8	1	10	1426	94.9%
14	100	100.5	10.5	7	0.5	30	2406	3.9%
15	100	200	1	8	0	50	0	0.0%
16	1	200	20	6	1	10	44	30.8%
17	50.5	100.5	1	7	0.5	30	6364	10.2%
18	100	200	20	8	0	10	420	20.3%
19	50.5	100.5	10.5	6	0.5	30	529	15.4%
20	1	1	1	6	1	10	35	0.3%
21	100	1	1	8	0	10	0	0.0%
22	50.5	100.5	10.5	7	0	30	218	24.3%
23	100	1	20	6	0	50	1	100.0%
24	1	200	1	6	1	50	128	63.2%
25	100	200	20	8	1	50	2413	15.7%
26	50.5	100.5	10.5	7	0.5	50	2249	15.8%
27	1	200	20	8	1	10	43	7.7%
28	1	1	1	8	0	50	18	100.0%
29	100	1	20	8	0	50	0	0.0%
30	50.5	100.5	20	7	0.5	30	1773	3.4%
31	1	200	1	8	0	10	142	31.0%
32	1	1	20	8	0	10	6	8.2%
33	100	200	1	6	0	50	12	100.0%
34	50.5	100.5	10.5	7	1	30	2644	0.6%
35	50.5	1	10.5	7	0.5	30	50	7.5%
36	1	1	20	6	0	10	1.6	2.2%
37	50.5	100.5	10.5	8	0.5	30	3609	7.6%
38	1	1	20	6	1	50	3	16.8%
39	1	200	20	6	0	50	1.4	100.0%
40	1	1	1	8	1	10	217	1.4%
41	50.5	100.5	10.5	7	0.5	30	2765	1.2%
42	100	1	1	8	1	50	39	83.0%
43	100	1	1	6	1	50	21	100.0%
44	50.5	100.5	10.5	7	0.5	30	2624	1.5%
45	50.5	200	10.5	7	0.5	30	4087	2.3%
46	1	200	1	6	0	10	0	0.0%
47	100	200	1	6	1	10	0	0.0%

Table S 11 Experimental table for the cross-BAL reaction generated by MODDE® and resulting TON and chemoselectivity determined by the ACQUITY UPC2®.

Factor	Value	Unit
Benzaldehyde	63	mM
Isobutyraldehyde	171	mM
Enzyme	20	uM
pH	6	
TPP	0.5	mM
DMSO	48	%
Predicted TON	2808	
Predicted chemoselectivity	76%	
Measured TON	219	
Measured chemoselectivity	79.5%	

Table S 12 Best condition predicted by MODDE® and the corresponding TONs and chemoselectivity for the cross-BAL reaction.

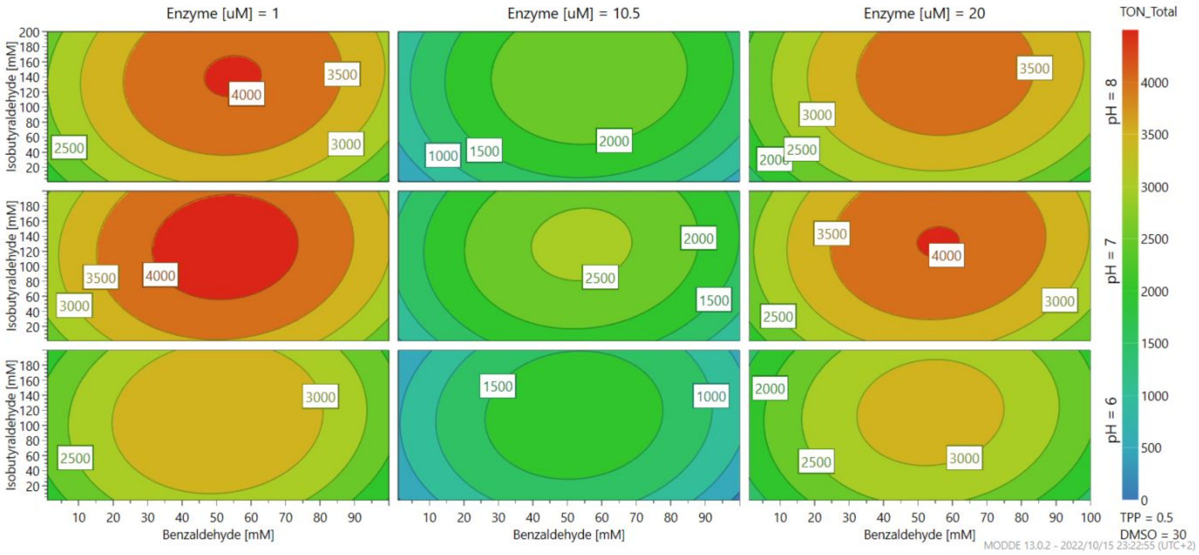

Figure S 4 Contour plot of TON for the cross-BAL reaction with TPP = 0.5 mM and DMSO = 30 % (v/v). TONs are represented by different colors. Variables include benzaldehyde concentration, isobutyraldehyde concentration, enzyme concentration and pH. Graph generated by MODDE®.

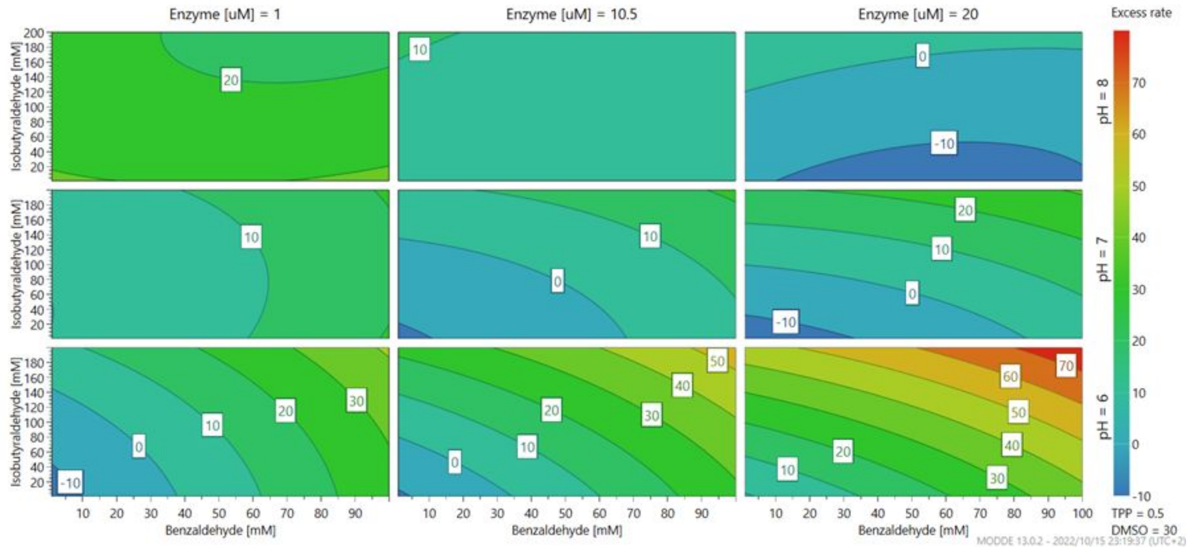

Figure S 5 Contour plot of chemoselectivity for the cross-BAL reaction at TPP = 0.5 mM and DMSO = 30 %. The resulting chemoselectivity is represented by different colors. Variables include benzaldehyde concentration, isobutyraldehyde concentration, enzyme concentration and pH. Graph generated by MODDE®.

BOA-2	Benz-aldehyde (mM)	Isobutyr-aldehyde (mM)	Enzyme (µM)	pH	TPP (mM)	DMSO (%)	Predicted TON	Measured TON	Predicted Chemoselectivity (%)	Measured Chemoselectivity (%)
Initial Data	1	1	1	6	1	10	-	35	-	0.7
	100	200	20	6	1	50	-	156	-	95.1
	50.5	100.5	10.5	7	0.5	30	-	2765	-	1.2
	100	1	20	8	1	10	-	27	-	9.5
	100	200	20	8	0	10	-	420	-	20.3
Cycle 1	100	200	20	6	0.9	50	668	93	53.9	98.1
	100	200	4.8	6	1	50	831	126	44.2	99.4
	10.9	200	20	6	1	50	851	55	43.2	74.1
	100	200	20	6	0	50	874	1	42.9	100.0
	100	200	20	6.2	1	50	664	272	55.6	92.3
Cycle 2	100	200	1	6	0	50	556	0	69.9	0.0
	100	1	20	6	0	50	587	0	61.6	100.0
	100	200	1	8	1	50	647	980	60.0	98.0
	1	200	20	6	0	50	549	2	65.3	91.4
	1	200	1	8	0	50	869	0	49.6	0.0
Cycle 3	100	200	20	8	1	50	529	2327	70.7	18.2
	100	1	20	6	1	50	345	2	72.7	82.3
	100	180.1	1	8	1	50	728	982	67.2	97.8
	1	1	20	6	0	50	411	0	64.7	100.0
	100	1	1	8	1	50	715	46	54.6	77.0
Cycle 4	100	200	2.9	8	1	50	944	2063	72.1	83.1
	100	200	1	7.8	1	50	865	1563	74.2	98.1
	100	200	4.8	8	1	50	997	2678	70.5	60.4
	1	1	20	8	0	50	579	0	55.8	0.0
	100	200	6.7	8	1	50	1042	3574	68.7	41.4
RSM	100	200	1	8	1	10		1426		94.9

Table S 13 Optimization process of the cross-BAL reaction by BOA-2 and comparison with the best result by response surface method (RSM).

	Benz-aldehyde (mM)	Isobutyr-aldehyde (mM)	Enzyme (uM)	pH	TPP (mM)	DMSO (%)	Predicted TON Total	Measured TON: TON (A)	Measured TON: TON (B)	Measured TON: TON Total	Predicted Chemo-selectivity (%)	Measured Chemo-selectivity (%)
initial data	1	1	1	6	1	10	-	17.35727	17.26081	34.61808	-	0.3
	100	200	20	6	1	50	-	3.851468	152.4331	156.2845	-	95.1
	50.5	100.5	10.5	7	0.5	30	-	1399.527	1365.079	2764.606	-	1.2
	100	1	20	8	1	10	-	14.51265	11.99853	26.51118	-	9.5
	100	200	20	8	0	10	-	167.1659	252.3901	419.556	-	20.3
cycle 1	70.3	200	14.3	6	0.4	50	997	1.197762	114.4604	115.6581	41.4	97.9
	10.9	200	20	7	1	50	960	202.3035	337.2094	539.5129	36.8	25.0
	100	200	1	6.6	1	50	951	257.4128	5183.695	5441.108	37.9	90.5
	100	20.9	20	6	0.1	50	999	1.351723	32.40401	33.75573	33.2	92.0
	100	200	1	7.2	0	50	1153	0	0	0	28.8	0.0
cycle 2	100	200	1	6.2	1	50	2445	2.477495	464.01	466.4874	65.1	98.9
	100	1	1	6	1	50	1770	0	0	0	52.9	0.0
	100	100.5	1	8	1	50	2133	132.6084	2196.384	2328.993	40.7	88.6
	100	200	1	6	1	10	1884	0	0	0	45.9	0.0
	1	200	1	6	1	50	1798	0	18.27914	18.27914	47.4	100.0

Table S 14 The detailed result of the cross-BAL reaction by BOA-1.

		BFD	PAL	cross-BAL: TON	cross-BAL: chemoselectivity
Response Surface Methodology		83.98	125.68	744.54	17.18
Bayesian Optimization	conventional	71.45	41.77		
	BOA1	302.30	4.35×10^{-5}	1160.69	1.26×10^{-6}
	BOA2	83.37	97.69	418.53	0.49

Table S 15 RMSE value of the final model including all the experimental data for each DoE method.

Material and Method

General information and instrumentation

All reagents and solvents, purchased from commercial sources, were used as received. All catalytic reactions were performed in 2 mL glass vials. Thin layer chromatography (TLC) was performed on Merck TLC Silica gel 60 F254 plates and visualized with ultraviolet light (254 nm), phosphomolybdic acid staining, or basic potassium permanganate staining. Column chromatography was performed on BUCHI Pure C-850 FlashPrep (Cyclohexane : ethyl acetate =1~5%, collected under 254 nm UV light). Catalytic experiments were analyzed on a Waters ACQUITY UPC2® or a Waters ACQUITY UPLC®. ^{1}H-NMR spectra were recorded at 500 MHz using a Bruker Avance instrument. Chemical shifts for ^{1}H-NMR spectra are reported in parts per million (ppm), and referenced to the solvent's residual peak ($CDCl_3$: 7.26 ppm). Multiplicities are represented as follows: s = singlet, d = doublet, t = triplet, q = quartet, hept = heptet, m = multiplet. Coupling constants (J) are expressed in hertz (Hz).

The water was purified with Milli-Q® Direct Water Purification System. SDS-PAGE was performed using Bio-Rad Mini-PROTEAN® TBE Precast Gels. Thermo Scientific™ Fresco™ 21 Centrifuge, Eppendorf™ Centrifuge 5920 R and Thermo Scientific™ Sorvall LYNX 6000 Superspeed Centrifuge were used for centrifugation.

Expression and purification of enzymes

The BAL enzyme used for catalysis was the wild type of BAL from *Pseudomonas fluorescens*. The BFD enzyme used for catalysis was the wild type of BFD from *Pseudomonas putida*. PAL used for catalysis was the C503S/C565S variant of BAL from *Anabaena variabilis*. The PAL gene was synthesized by Integrated DNA Technologies IDT on pET28a (NdeI/XhoI site) and sub-cloned into pRSF-Duet1vector.[1]

Buffers for purification. BFD and cross-BAL: resuspension buffer: Tris/HCl 50 mM, KCl 500 mM, pH 7.6; washing buffer: Tris/HCl 50 mM, KCl 500 mM, imidazole 50 mM, pH 7.6; elution buffer: Tris/HCl 50 mM, KCl 500 mM, imidazole 500 mM, pH 7.6; dialysis buffer: Tris/HCl 25 mM, NaCl 100 mM, pH 7.6. **PAL**: resuspension buffer: Tris/HCl 25 mM, NaCl 300 mM, pH 8.0; washing buffer: Tris/HCl 25 mM, NaCl 300 mM, imidazole 25 mM,

pH 8.0; elution buffer: Tris/HCl 25 mM, NaCl 300 mM, imidazole 250 mM, pH 8.0; dialysis buffer: Tris/HCl 25 mM, NaCl 300 mM, pH 8.0.

Protein production. The plasmids (see Supplementary Data) were transformed into competent cells (*E. coli* LEMO21 for PAL, *E. coli* BL21 (DE3) for BFD and cross-BAL) and grown on agar plates. A single colony was picked up from each plate and inoculated into 20 mL LB medium supplemented with selection antibiotic (50 µg/mL kanamycin for PAL, 100 µg/mL ampicillin for BFD and cross-BAL). The pre-cultures were incubated at 37 °C, 300 rpm overnight. The 20 mL pre-culture was used to inoculate the main culture (2 L Terrific Broth, TB) medium containing antibiotic (50 µg/mL kanamycin for PAL, 100 µg/mL ampicillin for BFD and cross-BAL) and incubated at 37 °C, 300 rpm until the OD reached 0.6 to 1.0. Then, 0.5 mM IPTG was added, and the culture was grown at 25 °C, 120 rpm overnight. The cells were harvested by centrifugation (4°C, 4000 g, 3 min). The cell pellet was resuspended in resuspension buffer which was supplemented with 1 mg/mL lysozyme and 0.05 mg/mL DNaseI, and incubated for 1 h (37 °C for PAL, 0 °C for BFD and cross-BAL). Sonication on ice (1s ON then 1s OFF, 60 cycles, repeat 3 times) was performed to fully lyse the cells. Then, the lysis solution was centrifuged (4°C, 50000 g for 30 min), and the clear supernatant was collected for protein purification.

Protein purification. To purify the proteins, 2 mL of Ni-NTA resin (column volume 1 mL) was added to a 10 mL column. The column was washed with water and equilibrated with the resuspension buffer. The clear supernatant of lysis samples was loaded via syringe through a filter (0.2 µm) to the column. After loading, the column was washed with 10x column volumes of washing buffer and subsequently eluted with 5x column volumes of elution buffer. Then, the eluent was dialyzed in a dialysis buffer (15 L) overnight twice. SDS-PAGE was performed to evaluate the molecular weight and purity of the protein samples. The pure protein solution was concentrated using Pierce™ Protein Concentrator PES and stored at -80 °C until usage.

.

Experimental procedure

BFD reaction: According to the experimental table, water, potassium phosphate buffer (50 mM, pH 6-8), DMSO (10-50%), TPP (0-1 mM), $MgSO_4$ (2.5 mM), benzaldehyde (1-100 mM), and BFD (1-20 µM) were added to a 2 mL glass vial to a total volume of 200 µL. The vials were shaken at 25 °C, 300 rpm for 48 h. Then the reaction mixture was extracted with an ethyl acetate solution (200 µL) containing biphenyl as an internal standard, and the organic layer was analyzed on UPC2. Conditions: CHIRALPAK® IA column 4.6 mm x 250 mm, 5.0 µm; mobile phase: 90% CO_2 and 10% isopropanol; flow rate: 2.5 mL/min; run time: 10 minutes. Peak areas were analyzed at 250 nm. The calibration curve and UPC2 chromatograms the of BFD reactions are displayed in **Figure S6**.

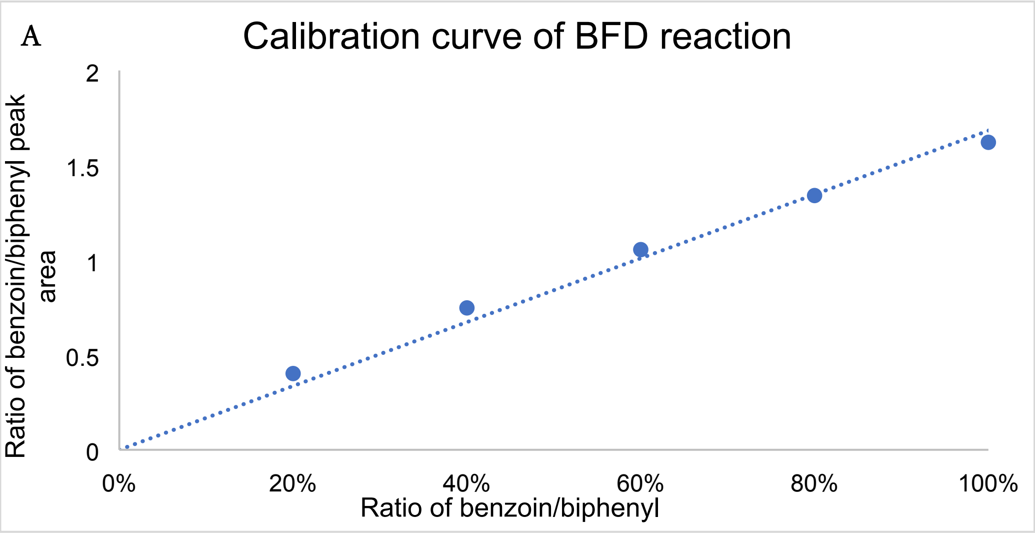

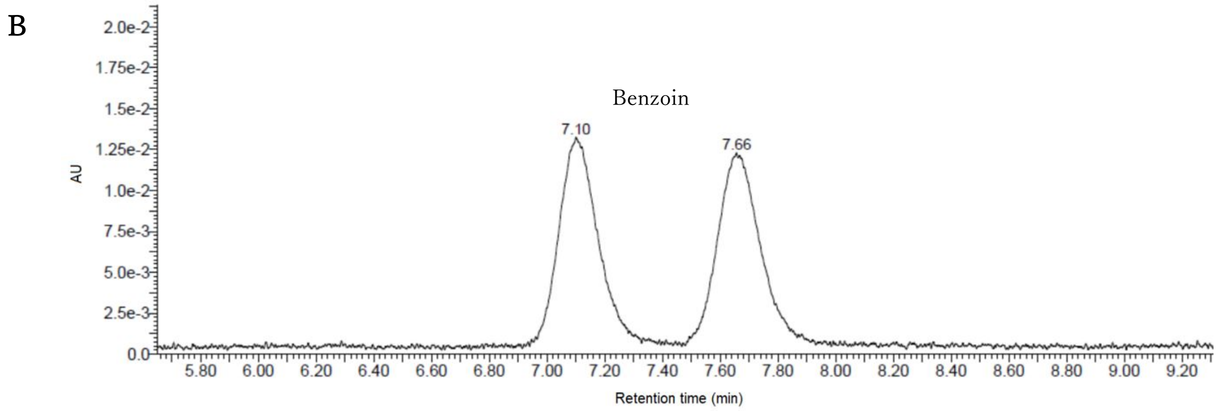

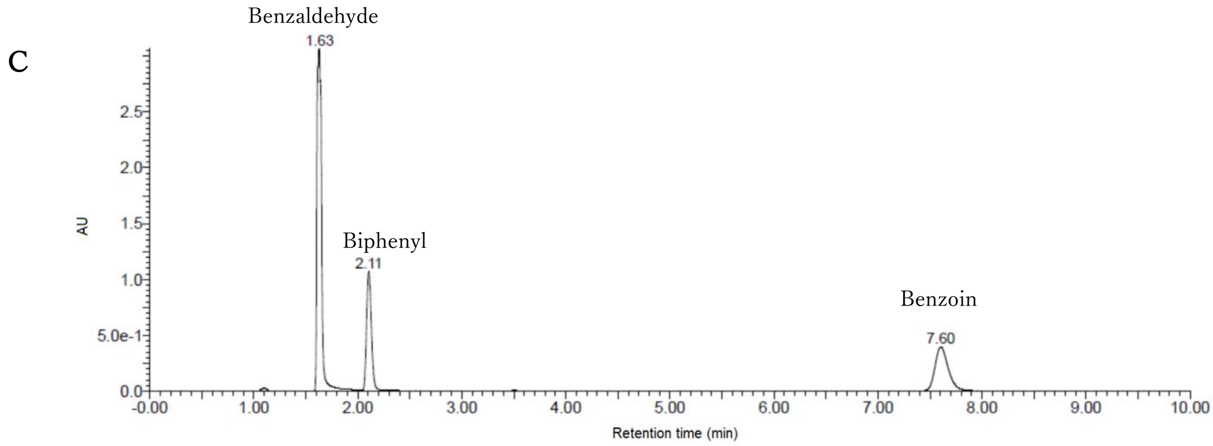

Figure S 6. A. Calibration curve for benzoin, with biphenyl used as internal standard. B. UPC2 chromatogram of racemic benzoin. C. UPC2 chromatograph of a typical BFD reaction. Peaks of benzaldehyde (starting material), benzoin (product), and biphenyl (internal standard) are displayed.

PAL reaction: According to the experimental table, water, NH_4OH/NH_4Cl buffer (pH 8-11), DMSO (5-50%), cinnamic acid (0.1-50 mM), and PAL (1-20 μM) were added to a 2 mL glass vial to a total volume of 200 μL. The vials were shaked at 30°C, 300 rpm for 48 h. Then the reaction mixture was mixed with 200 μL of internal standard solution (tryptophan) and then directly analyzed on reverse phase UPLC. Conditions: mobile phase: A, water + 0.1% TFA, B, acetonitrile + 0.1% TFA, B% = 1%-70%; flow rate: 0.55 mL/min; run time: 10 minutes. Peak areas were analyzed at 254 nm. Calibration curve and UPLC chromatography of PAL reactions are shown in **Figure S7**.

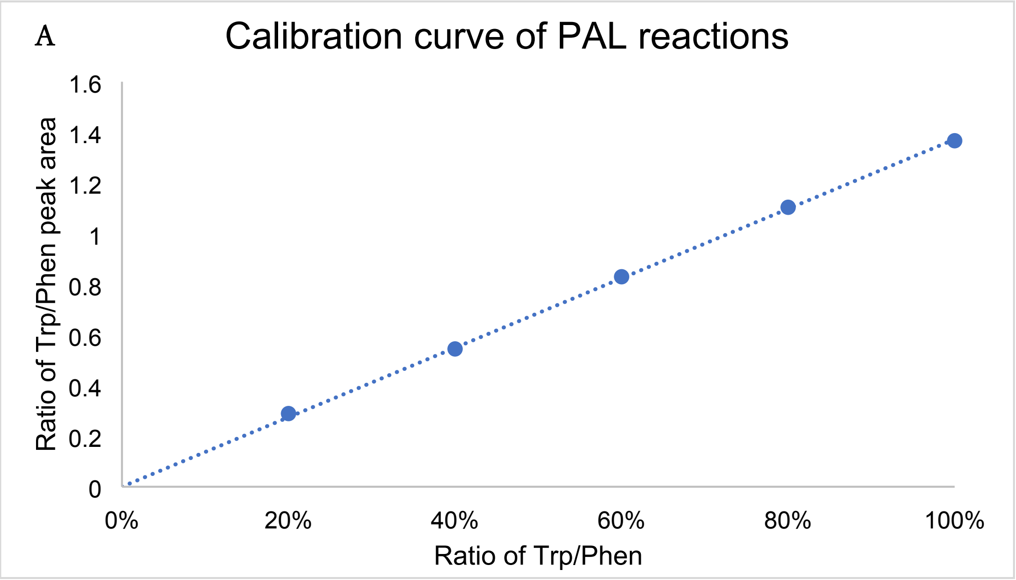

B

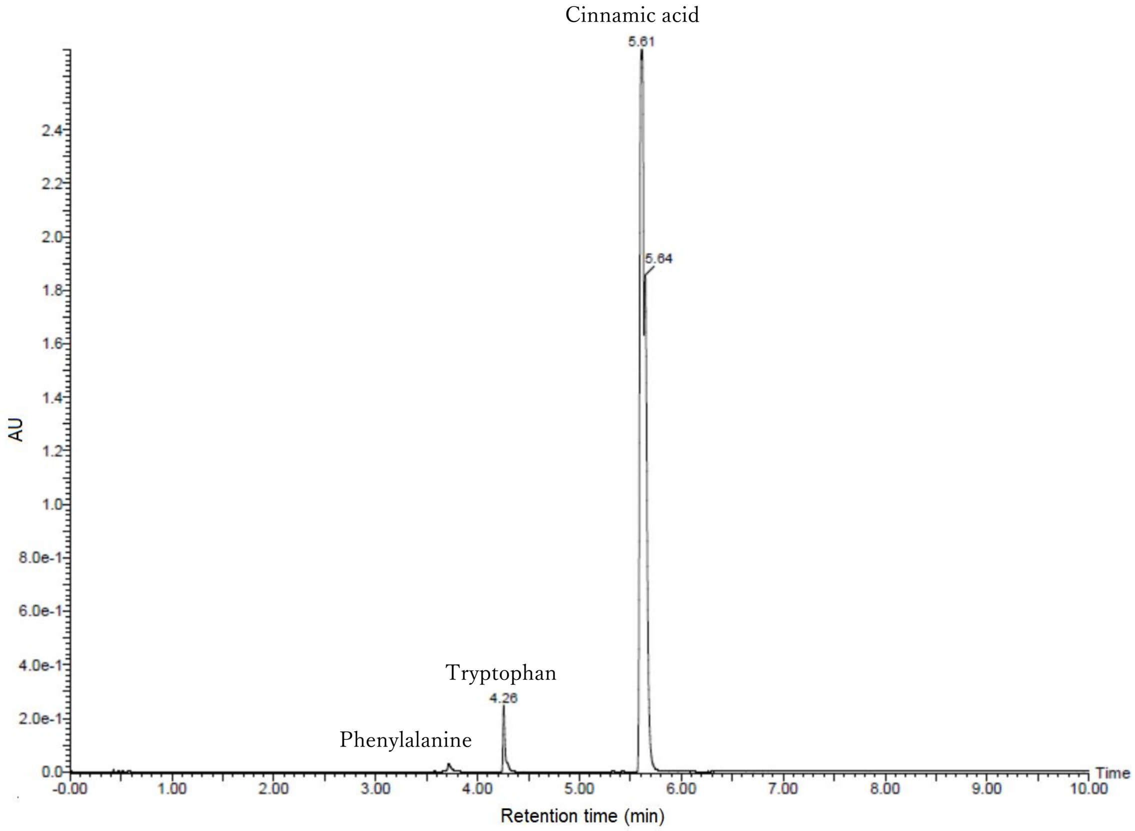

Figure S 7 A. Calibration curve of phenylalanine, with tryptophan as the internal standard. B. UPC2 chromatograph of typical PAL reaction. Peaks of cinnamic acid (starting material), phenylalanine (product), and tryptophan (internal standard) are shown.

Cross-BAL reactions: According to the experimental table, water, potassium phosphate buffer (50 mM, pH 6-8), DMSO (10-50%), TPP (0-1 mM), $MgSO_4$ (2.5 mM), benzaldehyde (1-100 mM), isobutyraldehyde (1-200 mM) and cross-BAL (1-20 μM) were added to a 2 mL glass vial to a total volume of 200 μL. The vials were shaked at 25 °C, 300 rpm for 24 h. Then the reaction mixture was extracted with 200 μL ethyl acetate solution of the internal standard (quinoline) and the organic layer was analyzed on UPC2. Conditions: CHIRALPAK® IA column 4.6 mm x 250 mm, 5.0 μm; mobile phase: 90% CO_2 and 10% ethanol; flow rate: 2.5 mL/min; run time: 10 minutes. Peak areas of Compound A were analyzed at 250 nm. Peak areas of Compound B were analyzed at 220 nm. Calibration curves and UPC2 chromatography of cross-BAL reactions are shown in **Figure S8**. For the calculation of chemoselectivity, the following equation was used:

$$Chemoselectivity = \frac{|Yield\ A - Yield\ B|}{Yield\ A + Yield\ B} \times 100\ \%$$

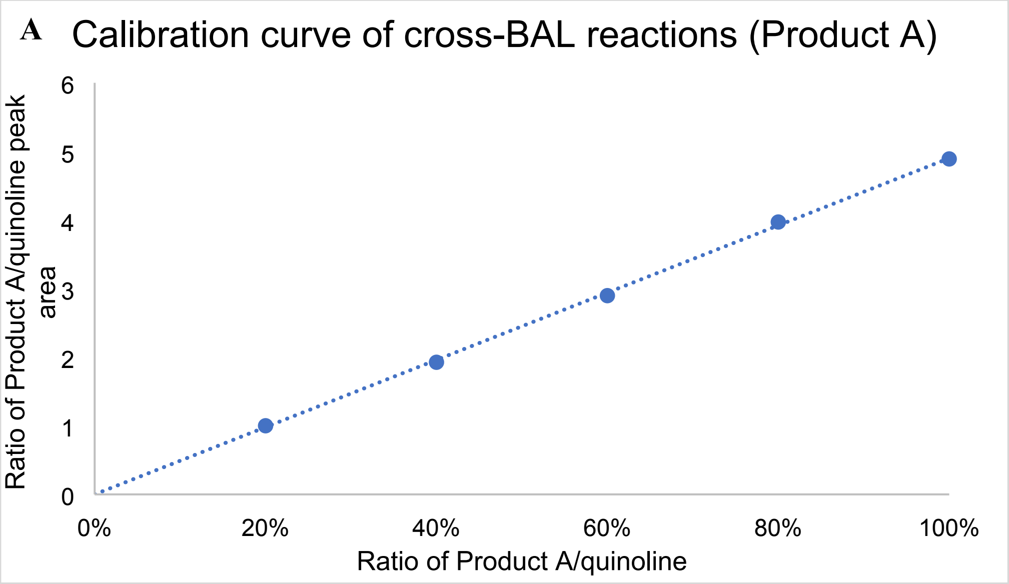

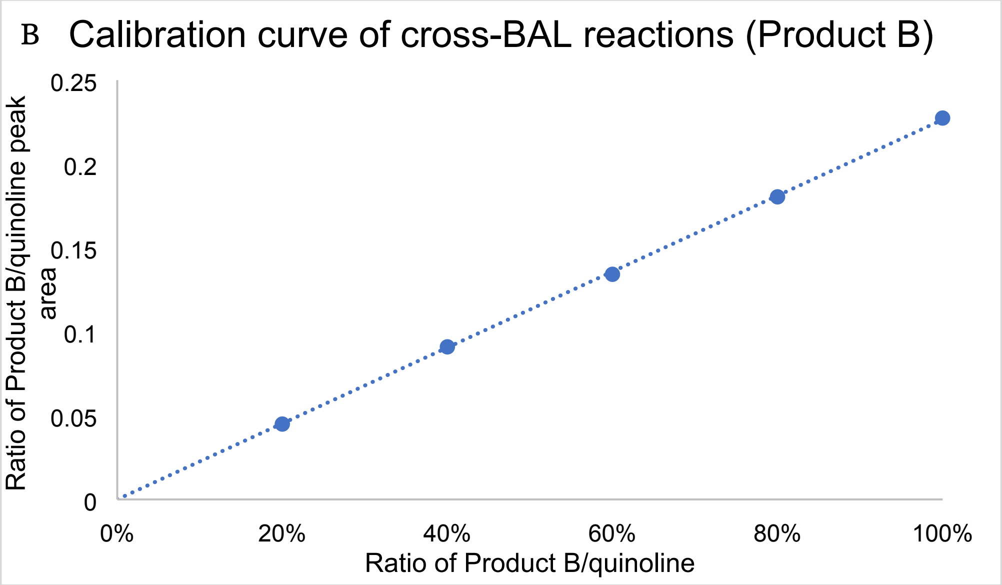

C

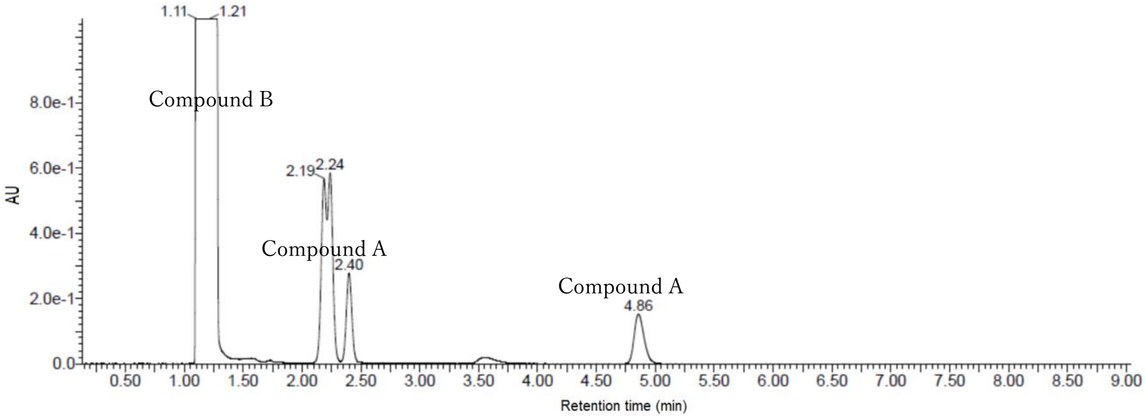

D

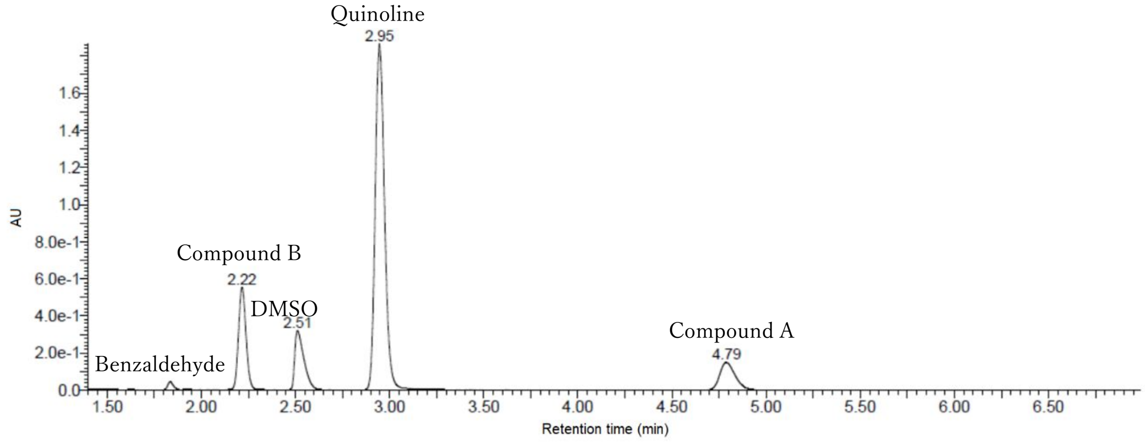

***Figure S 8** A and B. Calibration curve of Compound A and Compound B, with quinoline as the internal standard. C. UPC2 chromatograph of racemic benzoin. D. UPC2 chromatograph of typical BFD reaction. Peaks of benzaldehyde (starting material), Compound A and Compound B (products), and quinoline (internal standard) are shown.*

Generalized algorithm of BOA

Each experimental condition is normalized (min-max, from 0 to 1). For BOA-2, the measured FoM value is normalized (min-max, from 0 to 10). Then, these data are analyzed by Gaussian process regression. RBF kernel and the automatic relevance determination are used. The acquisition function is computed for each grid point of experimental conditions (resolution is 0.1). For BOA-1, the points in the vicinity (1 Euclidean distance) of the point adopted by the same iteration are removed from the candidate. The point whose acquisition function value is the biggest is taken as a condition to be tested.

Availability of the program

The BOA program was written with python, with GPy module[2].
It can be downloaded from https://github.com/TachibanaRyo-moroba/BOA
System requirements: Python 3.7 with additional modules (pandas, numpy, scipy, sklearn, GPy).

Synthesis

Synthesis of 2-hydroxy-3-methyl-1-phenylbutan-1-one (Compound A)[3]

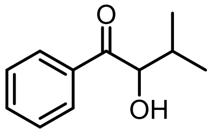

Benzaldehyde (630 µL, 6.2 mmol), triethylamine (512 µL, 3.7 mmol, 0.60 equiv.), 3-ethyl-5-(2-hydroxyethyl)-4-methylthiazolium bromide (156 mg, 0.62 mmol, 0.10 equiv.) and iso-butyraldehyde (2.00 mL, 18.7 mmol, 3.02 equiv.) were dissolved in ethanol (2.0 mL) and refluxed overnight at 85 °C under N_2. The reaction was quenched by addition of a saturated ammonium chloride solution (2 mL). The aqueous phase was extracted with ethyl acetate (3 x 15 mL), and the combined organic layers were dried over anhydrous $MgSO_4$. Filtration and concentration in vacuo afforded the crude product. Purification by chromatography yielded the racemic title compound (199 mg, 1.12 mmol, 18% yield) as a colorless oil. The product synthesized in this way was not pure. The main impurity was 4-hydroxy-2,5-dimethylhexan-3-one, the homo-coupled product of isobutyraldehyde, which was not easily separated by silica gel chromatography. Therefore, Compound A was only used to determine the retention time on UPC2.

Compound A used for quantification was synthesized by the enzymatic method. To a mixture of 80 mL potassium phosphate buffer (pH 8, final concentration 50 mM), cross-BAL (60 mg, 1 µmol), TPP (0.15 mM), and $MgSO_4$ (2.5 mM), a solution of 20 mL DMSO, benzaldehyde (153 µL, 1.50 mmol) and isobutyraldehyde (685 µL, 7.50 mmol) was added. The solution was stirred for 24 h at room temperature and afterwards quenched by addition of ethyl acetate (100 mL). After extraction with ethyl acetate (3 x 100 mL), the combined organic layers were washed with brine (50 mL). The organic phase was concentrated in vacuo after drying over anhydrous $MgSO_4$. Purification by chromatography yielded the pure title compound (26 mg, 0.15 mmol, 10% yield).

^{1}H NMR (500 MHz, Chloroform-*d*) δ 7.95 – 7.84 (m, 1H), 7.69 – 7.56 (m, 1H), 7.56 – 7.46 (m, 1H), 4.98 (d, *J* = 2.5 Hz, 1H), 2.18 – 2.09 (m, 1H), 1.17 (d, *J* = 6.9 Hz, 3H), 0.66 (d, *J* = 6.8 Hz, 3H).

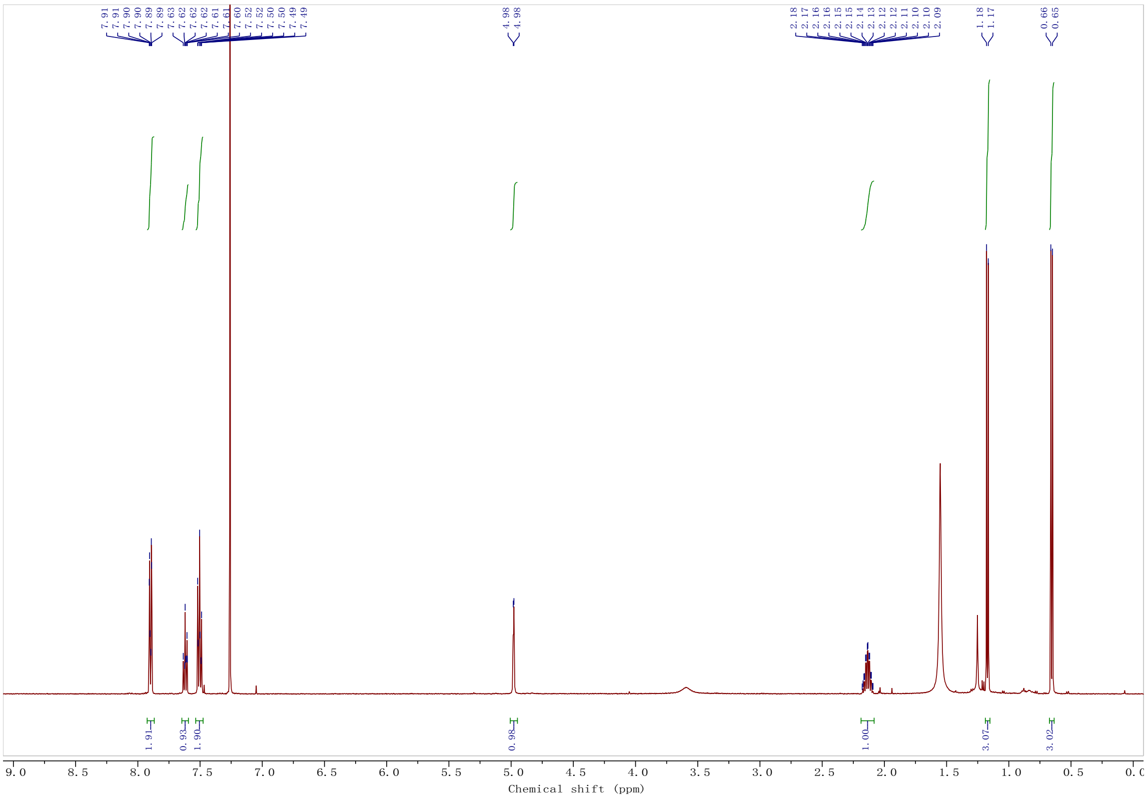

Synthesis of 1-hydroxy-3-methyl-1-phenylbutan-2-one (Compound B)[4]

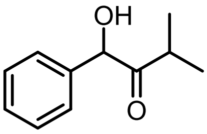

To a mixture of 6,7-dihydro-2-pentafluorophenyl-5H-pyrrolo[2,1-c]-1,2,4-triazolium tetrafluoroborate (18 mg, 0.05 mmol, 0.10 equiv.) and Cs_2CO_3 (16.29 mg, 0.05 mmol), benzaldehyde (50.82 μL, 0.5 mmol, 1.0 equiv.), isobutyraldehyde (685 μL, 7.5 mmol, 15 equiv.), and toluene (1 mL) were added under N_2. The reaction mixture was stirred at room temperature for 24 h, monitored with TLC, and then quenched with 2 mL distilled water and extracted with EtOAc (2 mL × 3). The combined organic layers were dried over anhydrous $MgSO_4$ and concentrated in vacuo. The residue was purified by column chromatography (EtOAc:cyclohexane = 1:20) to afford the racemic title compound as a colorless solid (27 mg, 0.15 mmol, 30% yield).

^{1}H NMR (500 MHz, Chloroform-*d*) δ 7.40 – 7.29 (m, 5H), 5.22 (d, *J* = 4.6 Hz, 1H), 4.37 (d, *J* = 4.6 Hz, 1H), 2.70

(hept, J = 6.8 Hz, 1H), 1.14 (d, J = 7.1 Hz, 3H), 0.84 (d, J = 6.7 Hz, 3H).

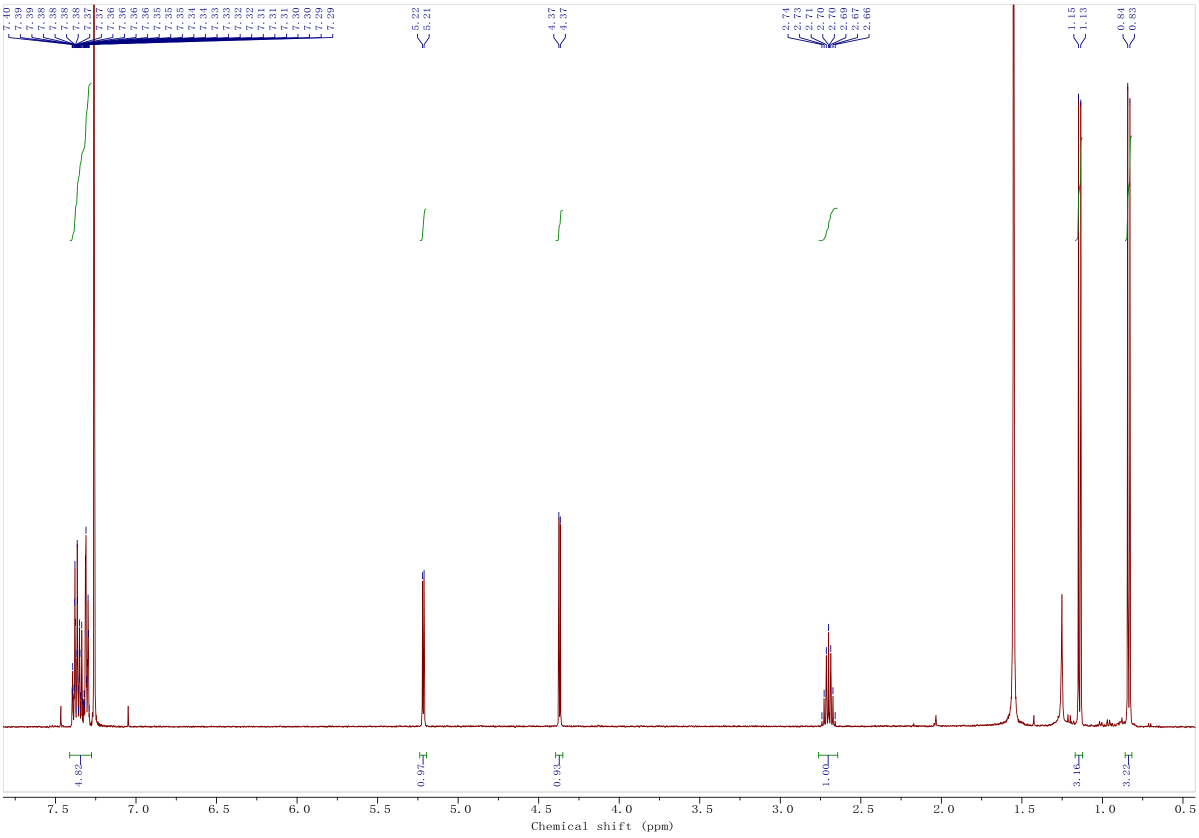

Supplementary References

(1) Wang, L.; Gamez, A.; Archer, H.; Abola, E. E.; Sarkissian, C. N.; Fitzpatrick, P.; Wendt, D.; Zhang, Y.; Vellard, M.; Bliesath, J.; Bell, S. M.; Lemontt, J. F.; Scriver, C. R.; Stevens, R. C. Structural and Biochemical Characterization of the Therapeutic Anabaena Variabilis Phenylalanine Ammonia Lyase. *J. Mol. Biol.* **2008**, *380* (4), 623–635. https://doi.org/https://doi.org/10.1016/j.jmb.2008.05.025.

(2) *GPy: A Gaussian process framework in python*. http://github.com/SheffieldML/GPy.

(3) Müller, C. R.; Pérez-Sánchez, M.; Domínguez de María, P. Benzaldehyde Lyase-Catalyzed Diastereoselective C–C Bond Formation by Simultaneous Carboligation and Kinetic Resolution. *Org. Biomol. Chem.* **2013**, *11* (12), 2000–2004. https://doi.org/10.1039/C2OB27344F.

(4) Jin, M. Y.; Kim, S. M.; Mao, H.; Ryu, D. H.; Song, C. E.; Yang, J. W. Chemoselective and Repetitive Intermolecular Cross-Acyloin Condensation Reactions between a Variety of Aromatic and Aliphatic Aldehydes Using a Robust N-Heterocyclic Carbene Catalyst. *Org. Biomol. Chem.* **2014**, *12* (10), 1547–1550. https://doi.org/10.1039/C3OB42486C.
